# Improved Osprey Optimization Algorithm Based on Two-Color Complementary Mechanism for Global Optimization and Engineering Problems

**DOI:** 10.3390/biomimetics9080486

**Published:** 2024-08-12

**Authors:** Fengtao Wei, Xin Shi, Yue Feng

**Affiliations:** School of Mechanical and Precision Instrument Engineering, Xi’an University of Technology, Xi’an 710048, China; 2220220077@stu.xaut.edu.cn (X.S.); 2230221283@stu.xaut.edu.cn (Y.F.)

**Keywords:** Improved Osprey Optimization Algorithm, L-good point set initialization strategy, adaptive dynamic adjustment, two-color complementary mechanism, simulation analysis, engineering problems

## Abstract

Aiming at the problem that the Osprey Optimization Algorithm (OOA) does not have high optimization accuracy and is prone to falling into local optimum, an Improved Osprey Optimization Algorithm Based on a Two-Color Complementary Mechanism for Global Optimization (IOOA) is proposed. The core of the IOOA algorithm lies in its unique two-color complementary mechanism, which significantly improves the algorithm’s global search capability and optimization performance. Firstly, in the initialization stage, the population is created by combining logistic chaos mapping and the good point set method, and the population is divided into four different color groups by drawing on the four-color theory to enhance the population diversity. Secondly, a two-color complementary mechanism is introduced, where the blue population maintains the OOA core exploration strategy to ensure the stability and efficiency of the algorithm; the red population incorporates the Harris Hawk heuristic strategy in the development phase to strengthen the ability of local minima avoidance; the green group adopts the strolling and wandering strategy in the searching phase to add stochasticity and maintain the diversity; and the orange population implements the optimized spiral search and firefly perturbation strategies to deepen the exploration and effectively perturb the local optimums, respectively, to improve the overall population diversity, effectively perturbing the local optimum to improve the performance of the algorithm and the exploration ability of the solution space as a whole. Finally, to validate the performance of IOOA, classical benchmark functions and CEC2020 and CEC2022 test sets are selected for simulation, and ANOVA is used, as well as Wilcoxon and Friedman tests. The results show that IOOA significantly improves convergence accuracy and speed and demonstrates high practical value and advantages in engineering optimization applications.

## 1. Introduction

Optimization can be formally defined as the process of identifying the optimal solution from among all feasible alternatives within a given problem. More specifically, it involves the maximization or minimization of a multidimensional function subject to a set of constraints. Optimization problems (OPs) are ubiquitous in real-world applications, encompassing areas such as business planning, ecological management, engineering design, and industrial control [1]. However, most real-world optimization problems are inherently complex and challenging to solve, with their complexity manifesting in multiple dimensions, such as multivariate, multi-objective, multi-constrained, nonlinear, multimodal, non-differentiable objective functions, and non-differentiable constraints. Mathematically, these problems are often classified as NP-hard, meaning that finding exact solutions requires substantial computational resources and may not be solvable in polynomial time. In this context, classical optimization methods such as gradient descent, branch-and-bound, cutting plane techniques, and dynamic programming often prove inadequate due to limitations such as susceptibility to local optima and high computational requirements in terms of both time and space. As a result, there is a pressing need for more efficient and robust methodologies to address complex OPs. To tackle OPs, optimization techniques can generally be categorized into three classes: exact methods, heuristic algorithms, and metaheuristics. The use of traditional exact optimization methods is typically impractical for complex problems due to their prohibitive computational demands. Heuristic algorithms, while more computationally feasible, tend to be highly dependent on specific problem characteristics. In contrast, metaheuristic algorithms are more advanced, employing problem-independent techniques, and are applicable to a wide range of optimization problems.

In the past 30 years, metaheuristic algorithms, as a stochastic approximate optimization method, have greatly overcome the above drawbacks and excelled in solving complex OP problems, which have attracted extensive attention and research. Among them, there are evolution-based metaheuristic algorithms, also known as evolutionary algorithms. They arise from the rules and processes of genetic and population evolution in nature and usually require iterative mutation, crossover, selection, and other related evolutionary operators. The most common are evolutionary planning (EP, 1966) [2], genetic algorithms (GA, 1975) [3], and differential evolution (DE, 1997) [4]. There are also physically based metaheuristic algorithms. These algorithms have a different optimization mechanism than evolutionary algorithms. Their search agents will exchange information and update the state in the search space according to physical rules. The most representative ones include Simulated Annealing (SA, 1983) [5], Golden sine algorithm (GSA, 2017) [6], and Chernobyl Disaster Optimizer (CDO, 2023) [7]. Secondly, there are also metaheuristics inspired by human concepts or behaviors. Examples include Cultural Algorithms (CA, 1994) [8], Harmony Search (HS, 2001) [9], Group Search Optimizer (GSO, 2006) [10], Student psychology-based optimization algorithm (SPBO, 2020) [11], mountaineering team-based optimization (MTBO, 2023) [12], etc. Finally, it is common to find population-based metaheuristic algorithms, which simulate the group behavior of a variety of animals and use information between groups of organisms as well as between them and their environment for communication and cooperation. Moreover, the goal of optimization is achieved by simple and limited interactions with experienced and intelligent individuals. The most classical ones are Particle Swarm Optimization (PSO, 1995) [13], Ant Colony Optimization (ACO, 1996) [14], Bat Algorithm (BA, 2012) [15], Sailfish Optimization Algorithm (SFO, 2019) [16], Remora Optimization Algorithm (ROA, 2021) [17], Waterwheel Plant Algorithm (WWPA, 2023) [18], Osprey Optimization Algorithm (OOA, 2023) [19], etc. Among them, OOA is a new swarm intelligence algorithm that is a fast, powerful, and high-performance optimization algorithm.

The algorithm emulates the natural foraging behavior of fish eagles, conferring a significant competitive edge over other heuristic algorithms. Although OOA possesses numerous advantages, it exhibits certain shortcomings when addressing complex engineering optimization problems, such as lower convergence accuracy and a tendency to become trapped in local optima. To enhance the overall performance of OOA, several researchers have introduced modifications aimed at overcoming these limitations. Yongliang Yuan et al. proposed the Attack-Defense Strategy-Assisted Osprey Optimization Algorithm (ADSOOA) [20], which integrates attack and defense phases, utilizing dynamic search parameters to strengthen global exploration capabilities and local exploitation strategies, thereby improving the algorithm’s convergence performance and avoiding local optima. Xiaodong Wen’s innovative multi-strategy fusion Improved Osprey Optimization Algorithm (SOOA) [21] significantly enhances the algorithm’s versatility and search efficiency through chaotic mapping initialization and a dynamically adjustable elite bootstrap mechanism. Yi Zhang et al. introduced an Improved Osprey Optimization Algorithm (IOOA) [22] for mixed-strategy optimization, which employs a Sobol sequence for initialization, a Weibull distribution step factor, and a perturbation strategy based on the principles of the firefly algorithm, achieving a highly diverse initial population and a balanced global exploration and local exploitation capability. The aforementioned literature represents improvements to OOA, enhancing its performance, yet further research is needed to better improve convergence accuracy and speed, as well as to achieve a balance between global search and local exploitation capabilities.

The aforementioned literature represents improvements to the Osprey Optimization Algorithm (OOA), which enhances its performance. However, further research is required to better improve convergence accuracy and speed, as well as to balance the global search and local exploitation abilities. Given this, the primary contributions of this paper are as follows:

Initialization: The population is initialized by combining logistic chaos mapping and the good point set method, ensuring a diverse initial population. The four-color theory is then applied to divide the population into four color groups (blue, red, green, and orange), aiming to balance the diversity of the population with the algorithm’s global search and local development capabilities.

Two-Color Complementary Mechanism: A two-color complementary mechanism is introduced in the algorithm design. The blue population maintains the original exploration strategy (OOA) to preserve fundamental effectiveness and stability. The red population adopts a heuristic strategy inspired by the behavior of the Harris Hawk to enhance the avoidance of local minima. The green population implements a strolling strategy to broaden the search boundaries and maintain population diversity. Lastly, the orange population combines the spiral search strategy and the firefly perturbation strategy to deepen the exploration of the solution space and perturb local optimal solutions, respectively, promoting the algorithm’s ability to escape from local optima and sustain long-term exploration vitality.

Comprehensive Validation: This paper conducts extensive validation through simulations of classic benchmark functions, CEC2020 and CEC2022 test sets, and statistical tests (ANOVA, Wilcoxon, and Friedman). The results show that the convergence accuracy and speed of IOOA are significantly improved while verifying its practical value and advantages in engineering optimization applications.

The article is structured as follows: Section 2 outlines the basic principles of OOA. Section 3 describes the details of the improved IOOA algorithm. Section 4 shows the results of testing and analyzing the new algorithm in comparison with the existing algorithms. Section 5 applies to the optimization examples of real engineering problems. Section 6 provides a detailed performance analysis of IOOA, summarizes the research results, and looks at future research directions.

## 2. OOA

Dehghani et al. were inspired by the fishing activities of fish eagles from lakes and proposed a novel heuristic optimization algorithm, Osprey Optimization Algorithm (OOA), in 2023. The algorithm simulates the process of fishing by fish eagles from lakes: the fish eagles capture their prey after detecting its location and then bring it to a suitable location to eat it. Therefore, in OOA, the location change process of the fish eagle population consists of two phases: the first phase is for the fish eagles to recognize the location of the fish and catch it (global exploration), and the second phase is for the fish eagles to bring the fish to a suitable location (local exploitation) after the first phase of the fish eagles’ recognition of the location of the fish and catching it (global exploration). This population intelligence algorithm achieves efficient exploration of the search space by simulating the fishing behavior of the fish eagle, which helps solve complex optimization problems.

### 2.1. Population Initialization

OOA is an intelligent population-based optimization algorithm inspired by the hunting behavior of ospreys in nature. Similar to other intelligent optimization algorithms, it performs random initialization of population operations in the search space by means of a population initialization formula:

(1) $$x_{i,j}=d_{j}+r_{i,j}\cdot \left(w_{j}-d_{j}\right),i=1,2,\cdots ,N,\;\;j=1,2,\cdots ,m$$

where $x_{i,j}$ is the individual, $d_{j}$ is the lower search limit, $w_{j}$ is the upper search limit, and $r_{i,j}$ is a random number between [0, 1].

### 2.2. Positioning and Fishing

In the OOA design, for each osprey, the locations of other ospreys in the search space with better objective function values are considered underwater fish. The first stage of population updating in OOA is modeled based on the simulation of the natural behavior of osprey hunting. The modeling of ospreys attacking fish results in significant changes in the positions of ospreys in the search space, which increases the exploratory power of the Osprey Optimization Algorithm in identifying optimal regions and escaping from local optima. In the design of OOA, for each osprey, the locations of other osprey in the search space with better objective function values are considered as underwater fish. The equation for the position of the fish corresponding to each osprey is shown below:

(2) $$Q_{i}=\left\{X_{k}|k\in \left\{1,2,\ldots ,N\right\}\wedge F_{k}<F_{i}\right\}\cup \left\{X_{best}\right\}$$

where *Q*_*i*_, is the set of locations of the *i*th fish eagle, *X*_*best*_ is the best candidate solution, *N* is the number of fish eagle populations, *F* and *F* are the objective function values corresponding to the *k*th and *i*th fish eagle, respectively.

The fish eagle randomly detects the position of one of the fish and attacks it. Based on the simulated movement of the fish eagle toward the fish, the new position of the corresponding fish eagle is updated using the following Equations (3) and (4).

(3) $$x_{i,j}^{P1}=x_{i,j}+r_{i,j}\cdot \left(H_{i,j}-I_{i,j}\cdot x_{i,j}\right)$$

(4) $$X_{i,j}^{t+1}=\left\{\begin{array}{c} x_{i,j}^{P1}\;{\;\;\;\;\;\;\;\;\;\;\;d}_{j}\leq x_{i,j}^{P1}\leq w_{j},\\ d_{j}\;{\;\;\;\;\;\;\;\;\;\;\;\;\;\;\;\;\;\;\;x}_{i,j}^{P1}<d_{j},\\ w_{j}\;\;\;\;\;\;\;\;\;\;\;\;\;\;\;\;\;\;\;\;x_{i,j}^{P1}<w_{j}, \end{array}\right.$$

(5) $$X_{i}=\left\{\begin{array}{c} Qx_{i}^{P1},\;{\;\;\;\;\;\;F}_{i}^{p1}<F_{i}\\ X_{i},\;\;\;\;otherwise \end{array}\right.$$

where $x_{i}^{P1}$ is the new position of the *i*th osprey in the first stage of the osprey-based optimization algorithm, $x_{i,j}^{P1}$ is its jth dimension, $H_{i}$ the fish selected by the jth osprey in the *i*th iteration, $H_{i,j}$ is its jth dimension and is a random number in the interval [0, 1], and $I_{i,j}$ is a random number from the set {1, 2}. If this new position is better, the previous position of the osprey is replaced according to Equation (5). where $F_{i}^{P1}$ is its objective function value.

### 2.3. Hunting and Feeding

After hunting a fish, the osprey takes it to a safe location to eat it. Phase 2 of the population update of OOA is based on the simulation of the natural behavior of the osprey to eat fish. In the design of the OOA, this behavior of the osprey is modeled. For each member of the population, a new random position is calculated as the “suitable position for eating fish” using Equations (6) and (7).

(6) $$x_{i,j}^{P2}=x_{i,j}+\frac{d_{j}+r\cdot \left(w_{j}-d_{j}\right)}{t},i=1,2,\cdots ,N,j=1,2,\cdots ,m,t=1,2,\cdots ,T$$

(7) $$X_{i,j}^{P2}=\left\{\begin{array}{c} x_{i,j}^{P2}\;{\;\;\;\;\;\;\;d}_{j}\leq x_{i,j}^{P2}\leq w_{j},\\ d_{j}\;\;\;\;\;\;\;\;\;\;\;\;\;\;x_{i,j}^{P2}<d_{j},\\ w_{j}\;\;\;\;\;\;\;\;\;\;\;\;\;\;x_{i,j}^{P2}<w_{j}, \end{array}\right.$$

(8) $$X_{i}=\left\{\begin{array}{cc} Qx_{i}^{P2}, & F_{i}^{P2}<F_{i}\\ X_{i}, & otherwise \end{array}\right.$$

where $X_{i}^{P2}$ is the new position of the *i*th osprey based on OOA, $x_{i,j}^{P2}$ is its *j*th dimension, $x_{i}^{P2}$ is the optimal value of its objective function, *t* is the number of iterations, and *T* is the maximum total number of iterations. Then, if the objective function value is improved at this new position, it will replace the previous position of osprey according to Equation (8).

## 3. IOOA

In this section, IOOA will be introduced in detail, which is an efficient search algorithm that incorporates multiple optimization strategies based on the OOA. In the initialization phase of the IOOA, the initial populations generated by the strategy of fusing the logistic mapping with the set of good points in order to produce a diverse and uniformly distributed initial populations; secondly, drawing on the well-known conclusions of the Graph Theory, the four-color theorem divides the populations into four parts, which are the red group, orange group, blue group, and green group, and the focus of the search methods of different groups is different, and the four groups of populations will use the two-color complementary mechanism to improve the position update, and these strategies together constitute the core search mechanism of the IOOA algorithm.

### 3.1. L-Good Point Set Initialization Strategy

In the initialization phase of the OOA, the population is constructed using the L-good point set method, which is an initialization strategy that combines the logistic chaotic mapping [23] and the good point set method [24]. The L-good point set method carefully selects or constructs a series of “good points” in the solution space through specific mathematical constructs (e.g., based on prime numbers, cosine function, etc.), which usually have good spatial distribution characteristics and can cover the key regions of the solution space, avoiding the problem of concentrated or sparse distribution of points that may be caused by random initialization. Logistic mapping, as a kind of chaotic mapping, is capable of generating sequences with a high degree of randomness and long-term unpredictability, which helps expand the search range of feasible solutions, balancing the trade-off between local exploitation performance and the global search capability of the algorithm.

#### 3.1.1. Good Point Set Method

Good point set theory is a branch of mathematics that focuses on the set formed by all points that are a certain distance away from a point in a Euclidean space. This theory has a wide range of applications in geometry, topology, and number theory. An intuitive way to choose n points from X to maximize the likelihood that it contains an optimal point for a thesis X when nothing is known about its traits is to take n points and make it the most uniformly distributed in X. Because of the most uniform distribution, the probability of taking the optimal point is maximized. To be uniform, minimizing deviation in number theory is uniformity, and this problem of minimizing deviation can be explained by using good points in number theory.

In practice, the following three methods are usually used:

Exponential sequence methods: $r_{k}=\{e^{k},1\leq k\leq m\}$;

Square root sequence method: $r_{k}=\sqrt{p_{k}},1\leq k\leq m$, $r_{k}$ are mutually unequal prime numbers;

Split-circle domain method: $r_{k}=2\cos\frac{2\pi k}{p},1\leq k\leq m$, *p* is the smallest prime number satisfying the condition (*p* − 3)/2 ≥ *m*.

The principle of good point sets used in this paper is as follows:

Let $G_{s}$ be a unit cube in s-dimensional Euclidean space if $r\in G_{s}$, of the form.

(9) $$P_{n}\left(k\right)=\left\{\left(\left\{r_{1}^{\left(n\right)}\cdot k\right\},\left\{r_{2}^{\left(n\right)}\cdot k\right\},\cdots ,\left\{r_{s}^{\left(n\right)}\cdot k\right\}\right),1\leq k\leq n\right\}$$

Its deviation $\varphi_{\left(n\right)}$ satisfies $\varphi_{\left(n\right)}=C\left(r,\varepsilon \right)n^{-1+\varepsilon }$, where $C\left(r,\varepsilon \right)$ is a constant related only to *r* and *ε* (*ε* is an arbitrary positive number), then $P_{n}\left(k\right)$ is said to be the set of good points, and r is the good point. $\left\{r_{s}^{\left(n\right)}\cdot k\right\}$ stands for taking the fractional part, *n* denotes the number of points, and *r* = {2 cos(2*πk*/*p*), 1 ≤ *k* ≤ *s*} (*p* is the smallest prime number satisfying (*p* − 3)/2 ≥ *s*). Map this to the search space as follows:

(10) $$x_{i}\left(j\right)=\left(ub_{j}-lb_{j}\right)\cdot \left\{r_{j}^{\left(i\right)}\cdot k\right\}+lb_{j}$$

${ub}_{j}$ and ${lb}_{j}$ denote the upper and lower bounds of the *j*th dimension.

The initialization problem of IOOA is essentially how to search a larger space using a finite number of individual ospreys. If the initial population is randomly generated, it cannot traverse the various conditions in the solution space. Utilizing the construction method of the theory of good point sets, its computational accuracy is independent of the number of dimensions, so drawing on the method of good point sets to uniformly design the initial osprey population can overcome the shortcomings of the above methods and can produce an initial population with better diversity.

#### 3.1.2. Logistic Chaotic Map

Logistic chaotic mapping demonstrates significant benefits in population initialization by virtue of its ability to generate highly stochastic and long-term unpredictable sequences. This study integrates logistic chaotic mapping with the preferred point set technique with the aim of optimizing the quality of the algorithm’s starting solution. The mapping formula is as follows:

(11) $$x_{i}^{t+1}=ax_{i}^{t}\left(1-x_{i}^{t}\right)$$

Here, $x_{i}^{t}$ and $x_{i}^{t+1}$ denotes the current and next iteration states of the *i*th variable within the interval [0, 1], respectively; the parameter *a*, governing the degree of chaos, ranges within (0, 4], with *a* = 4 marking full chaotic behavior. The chaotic trajectory spans the interval (0, 1).

#### 3.1.3. L-Good Point Set Method

In optimization algorithms, the quality and diversity of the initial population are crucial to the algorithm’s optimization search ability and convergence performance. In order to enhance the global search ability and convergence performance of the algorithm, logistic mapping is fused with the good point set method—L-good point set method. The main principle of this strategy is to combine the chaotic sequences generated by logistic mapping with the good point set strategy during the initialization process, implying that each element of the chaotic sequences is mapped onto the unit sphere to form a good point set. This combination produces an initial population that is both stochastic and maintains structure by combining the randomness of the chaotic dynamical system with the orderliness of the other structures to improve search efficiency and global search capability. This strategy not only enhances the optimization ability and search efficiency of the whole population but also speeds up the convergence of the algorithm. In this way, the optimization algorithm is provided with a stronger search ability in the initialization stage and better solves the actual optimization problem.

**Figure 1:**
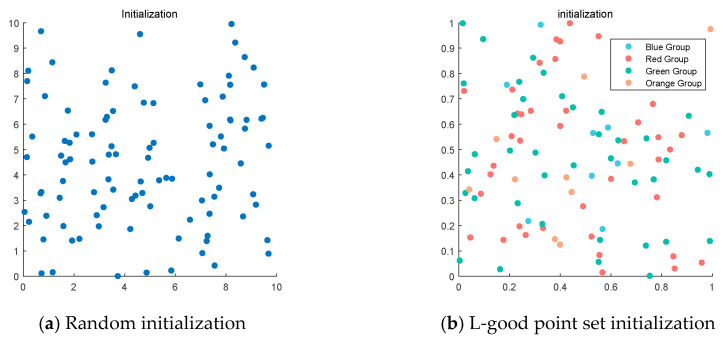
Comparison of population initialization.

This strategy can effectively avoid the algorithm’s blind search, reduce the solution efficiency, and lay the foundation for improving the solution accuracy and accelerating the convergence speed. Figure 1 clearly shows the comparison between the fish eagle population before and after initialization.

### 3.2. A Two-Color Complementary Strategy Based on the Four-Color Theorem

OOA, as an emerging bio-heuristic optimization algorithm, has a strong ability to find the optimal and certain search accuracy, but any algorithm has its limitations, and the OOA is no exception. In some complex optimization problems, different regions of the solution space may require different search strategies. The population of OOA is not classified, and all individuals may follow the same search rules and step sizes, which may limit the algorithm’s adaptability to different regions and exploration efficiency. A single search strategy may perform poorly in dealing with problems with multiple local optimal solutions, which may easily cause the algorithm to prematurely converge to a non-global optimal solution, especially when there is no effective mechanism to promote population diversity or escape from local optimality. To address these shortcomings of OOA, this paper adopts a two-color complementary strategy based on the four-color theorem [25] for fish eagle populations.

#### 3.2.1. Stock Grouping Adjustment Mechanism

In OOA, the execution of the same search strategy by all populations may bring certain limitations. When encountering problems with more solution spaces, it is difficult to fully search the global solution space due to fewer population searching methods, and when multiple local optimal solution problems are encountered, it is difficult to jump out of the local optimal solution due to the single searching method. In order to overcome this problem, this paper draws on the four-color theorem in number theory to introduce a grouping adjustment mechanism, which divides the population into four-color populations composed of two pairs of complementary colors, which are the red group and the green group as well as the blue group and the orange group, respectively.

Initially, set the mathematical expression for the ratio of the red group to the green group:

(12) $$P_{red}^{\prime }=0.4$$

(13) $$P_{green}^{\prime }=0.4$$

As the main populations in the IOOA algorithm, the red group and the green group act as the main exploring group, whose main task is to carry out extensive and in-depth exploration in the solution space and work on discovering new solution regions. Through extensive exploration, the exploration group helps prevent the algorithm from falling into local optimality too early, provides fresh solution ideas for the whole algorithm, and increases the probability of finding the global optimal solution. Initially, set the mathematical expression for the ratio of the blue group to the orange group:

(14) $$P_{blue}^{\prime }=0.1$$

(15) $$P_{orange}^{\prime }=0.1$$

**Figure 2:**
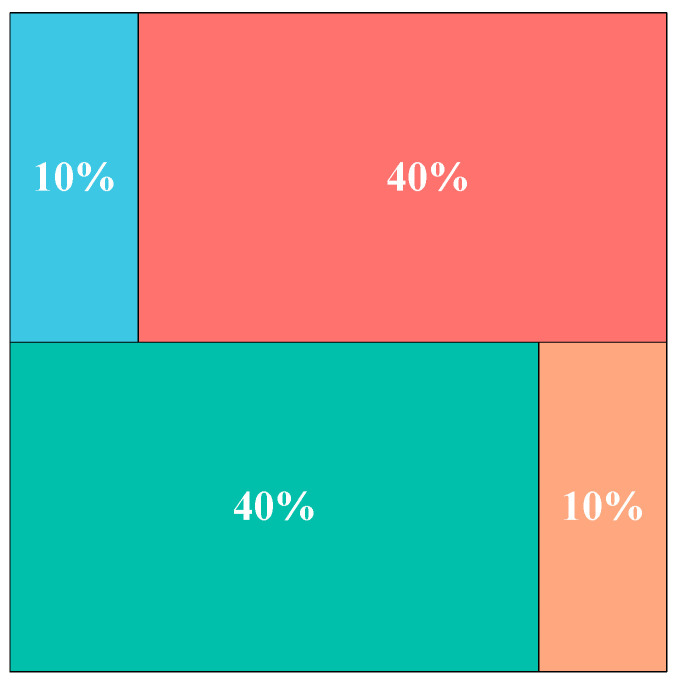
Grouping based on the four-color theorem.

The blue group and the orange group are in development in the IOOA algorithm, whose main responsibility is to perform fine search and local optimization around the found better solution regions. By focusing on the refinement of local regions, the development group helps improve the quality of the solutions and promotes the algorithm to converge to higher quality solutions, especially for those complex optimization problems that contain multiple local minima. Implementing such a population grouping tuning strategy performs different strategies by grouping the overall population while improving the overall performance and optimization search efficiency. Figure 2 illustrates the grouping based on the four-color theorem in the population.

#### 3.2.2. Two-Color Complementary Mechanism

In OOA, a certain global search capability is maintained at the initial stage by randomly selecting “prey” and a large exploration step size, which helps cover a wide search space. However, if the initial setup is poor or the adaptation is complex over a wide area, the exploration phase may not be sufficient to fully traverse all critical regions, and as the iteration progresses, especially in the second phase, the algorithm shifts to rely more on searching in the vicinity of the current optimal solution, and the local search capability increases. While this helps refine the quality of the solution, it may also fall into a local optimum by shrinking the search range too quickly. To solve the above problem, a two-color complementary mechanism is introduced, where the current population is divided into red and green groups, blue group, and orange group according to the above grouping based on the four-color theorem, and different update strategies are adopted for the populations of different color groups. Specifically, for the red and green groups, as the main exploratory groups, a larger variation step or a more aggressive search strategy can be adopted to skip the neighborhood of existing solutions and find the potential global optimal solution. For the blue and orange groups, a smaller variation step size or a local search strategy can usually be adopted to gradually improve the existing solutions. At the same time, this paper introduces a two-color complementary mechanism to strengthen the correlation between populations by adopting the method of mutual exchange between the exploring and exploiting groups within the group, such that the different updating strategies of multiple populations can help to balance the exploration and utilization of individuals in the search space, improve the global search performance of the algorithm, and thus search for the optimal solution more efficiently.

Determine the size of the red, green, blue, and orange group populations:

$N_{red}=\left[N\cdot P_{red}^{\prime }\right]$: red group population size;

$N_{green}=\left[N\cdot P_{green}^{\prime }\right]$: green group population size;

$N_{blue}=\left[N\cdot P_{blue}^{\prime }\right]$: blue group population size;

$N_{orange}=\left[N\cdot P_{orange}^{\prime }\right]$: orange group population size.

**Figure 3:**
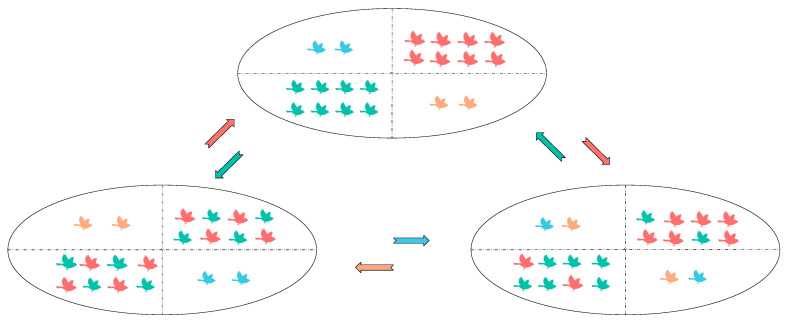
Two-color complementary mechanism.

After randomly sorting according to fitness, the population is divided into four parts, and the population exchange within the exploration group and within the exploitation group is realized at the same time. The following is the design of the specific exchange method:

$p_{0}=0.5$: the initial intra-group exchange probability;

δ=0.02: the decay rate of the exchange probability in each generation;

$p_{min}=0.2$: the minimum exchange probability;

pt=max⁡p0×(1−δ)^t,pmin: the formula for the decay of the exchange probability, t is the number of iterations. The specific two-color complementary exchange idea is shown in Figure 3.

IOOA employs a two-phase strategy framework that aims to balance global exploration with local exploitation capabilities through the two-color complementary mechanism. This framework is divided into two core phases: the initial broad exploration (exploration phase) aims to explore the vast potential of the solution space, while the subsequent deep optimization (exploitation phase) focuses on escaping the local optimum and refining the solution. Based on this framework, this paper implements a differentiation strategy for each group of populations through the color population division method to enhance the global search efficiency and convergence accuracy of the algorithm.

Specifically, the blue population follows the OOA framework and retains the original exploration and exploitation strategy, in which the exploration phase encourages a broad search of the solution space, while the exploitation phase focuses on the detailed optimization in the vicinity of the existing better solutions, which embodies the core idea of the traditional bio-inspired algorithm.

The red population, on the other hand, has made strategic innovations in the framework of OOA, especially in the exploitation phase, where the HHO is introduced. This strategy demonstrates the optimization and extension of the traditional algorithm by dynamically adjusting the exploration behavior and development behavior of individuals based on the current iterative progress and stochastic factors, which effectively facilitates the escape from the local optimum.

The strategy of the green population achieves an enhancement of the flexibility of the search strategy by dynamically adjusting the intensity of the random walks while maintaining the iterative exploration. Specifically, depending on the different stages of the algorithm operation, the green population is able to flexibly switch between global exploration and local enhancement, which enhances the efficient coverage of the solution space by exploiting the time-dependent neighborhood search radius variation.

As for the orange population, the optimized spiral search strategy is adopted in the exploration phase, which utilizes the time-decay parameter and randomized spiral motion to not only broaden the search path but also increase the randomness of the exploration process. As for the exploitation stage, the orange population borrows the firefly perturbation strategy, which achieves the fine adjustment and optimization of the local optimal solution by simulating the attraction and repulsion behaviors between fireflies, demonstrating a high degree of strategy innovation and optimization ability.

In summary, by implementing strategy customization for each color population separately, IOOA not only embodies the diversity and flexibility of the algorithm design but also effectively improves the global exploration efficiency and local convergence accuracy of the algorithm in solving complex optimization problems by integrating a variety of bio-inspired strategies.

The mathematical expressions and search logic of these various population adoption strategies are explained in detail below.

Harris Hawk-inspired strategy:

The strategy of the red population employs the escape energy mechanism in the Harris Hawk optimization algorithm [26] in the second phase to facilitate further optimization of the solution, especially in solving the local optimum trap problem. The core of the strategy in this phase is to simulate the collaborative hunting behaviors exhibited by Harris Hawks in nature and their dynamic flight strategies when escaping from predators as a way to enhance the algorithm’s exploratory capability and ability to jump out of local optima.

The formula for the energy decay factor E_1_ is shown below:

(16) $$E_{1}=2\left(1-\frac{t}{T}\right)$$

denotes the gradual decay of the Harris Hawk’s “escape energy” as the number of iterations, *t*, increases, simulating a gradual shift from extensive exploration to more focused development of the algorithm over time.

The escape energy E formula is shown below:

(17) $$E=E_{1}\left(2r-1\right)$$

where *r* is a random number between [0, 1]. The escape energy E determines the strength of the escape, which can be either positive (exploration) or negative (exploitation), enhancing the randomness and flexibility of the strategy.

Depending on the random number q between the escape energy *E* and [0, 1], the Harris Hawk’s attack is determined, one encouraging exploratory behavior away from the current solution and the other exploitative behavior with more fine-tuning toward the globally optimal solution.

When the escape energy $\left.\left|E_{1}\right.\right|\geq 1$, the Harris Hawk performs the exploration behavior, and the update rule formula is shown below:

The nonlinear inertia weights $\omega_{2}$ are calculated as shown below:

(18) $$X_{new}=\left\{\begin{array}{cc} X_{current}-E\left|\left.X_{random}-2r\cdot X_{current}\right|\right., & q<0.5\\ \left(X_{Best}-\bar{X_{population}}\right)-E\left|\left.X_{Best}-X_{current}\right|\right., & q\geq 0.5 \end{array}\right.$$

When the escape energy $\left.\left|E_{1}\right.\right|<1$, the Harris Hawk performs the exploitation behavior, updating the rule equation as shown below:

(19) $$X_{new}=X_{current}+E\left|\left.X_{Best}-X_{current}\right|\right.$$

The second-stage strategy of the red population achieves a smooth transition from extensive exploration to local fine exploitation by incorporating the biobehavioral characteristics of the Harris Hawk, especially in dealing with the local optimum problem, which demonstrates a unique strategic advantage. Through the dynamically adjusted escape energy and flexible exploration and exploitation mechanism, the algorithm is able to effectively jump out of the local optimum trap and continuously explore and optimize the solution space, thus improving the overall optimization performance.

Random walk strategy:

The strategy design of green population aims to balance the exploration and exploitation capabilities of the algorithm through two phases—the global exploration phase and the local exploitation phase—in order to efficiently search the solution space and eventually converge to a high-quality solution during the optimization process, which is exactly what the random walk strategy [26] can satisfy. The following is a detailed analysis of these two phases of the strategy and the interpretation of the relevant mathematical formulas:

The formulas for the global exploration phase are described below: In the early stage of IOOA, the search range is wide, and an exponential decay function is used to control the rapid narrowing of the exploration range with the increase in the number of iterations t, encouraging a fast coverage of the solution space, the exponential decay function whose formula is shown below:

(20) $${NF}_{\left(t\right)}=0.2e^{-\frac{t}{T\times 0.3}}$$

In IOOA, a random vector is used in this paper for randomly selecting the search direction to increase the randomness and diversity of the search, and the random perturbation function whose formula is shown below:

(21) $$MD=2\left(U\left(0,1\right)-0.5\right)$$

where *U* (0,1) represents a uniformly distributed random variable.

The position update formula for the global exploration phase is as follows:

(22) $$X_{newglobal}=X_{Best}+{NF}_{\left(t\right)}\cdot MD$$

When t≥M×0.3, the algorithm starts to enter a localized development phase based on the increase in the number of iterations *t*. As the iterations go deeper, the perturbations get smaller and smaller, which helps make fine adjustments rather than large jumps. The formulas for the local development phase are introduced as follows:

(23) $$X_{new}=X_{Best}+\left(ub-lb\right)\frac{rand\left(1,dim\right)}{\left(t-0.3M\right)}$$

This formulation focuses on optimizing in the vicinity of the discovered better solutions by reducing the magnitude of the perturbations. The strategy of green populations combines the advantages of global and local search by dynamically adjusting the exploration range and the magnitude of the perturbations, which both achieves a broad exploration of the solution space and ensures efficient local optimization after discovering the potentially optimal region.

### 3.3. Optimize Spiral Search Strategy

The strategy of orange population is designed around two core phases, the exploration phase and the exploitation phase, each of which employs a unique bio-heuristic strategy to balance the exploration and exploitation capabilities of the algorithm, aiming to improve the quality of search efficiency and solution. The search strategy [27] used in the first phase of the orange population is an optimized spiral search strategy, which extensively explores the solution space by simulating a spiral motion with the aim of jumping out of the local optimum and discovering new solution regions.

The formulation of the global exploration phase with respect to the orange population is presented as follows:

(24) $$\alpha =2(1-\frac{t}{T})$$

α is a linear decay parameter that decreases with the number of iterations *t*, reflecting the gradual reduction in the exploration range over time.

(25) $$l=2\pi \left(1-\frac{t}{T}\right)\cdot rand$$

*l* is the spiral angle, which combines the current iteration ratio $\frac{t}{T}$ and a random number to introduce a randomization factor to increase the diversity of the search. Rand represents a random number between 0 and 1.

To further increase the randomness of the search, a deflation factor *A* is introduced by combining the linear decay parameter *α* and another random number with the following equation:

(26) A=2α·rand

Combining spiral motion and random perturbations to form a new positional candidate solution. The formula is as follows:

(27) $$X_{new}=X_{Orange}+A\left(\left|\left.X_{Best}-X_{Orange}\right|e^{2l}\cos\left(l\right)+0.1\left(ub-lb\right)\left(rand\left(1,dim\right)-0.5\right)\right.\right)$$

### 3.4. Firefly Spoiler Strategy

Based on the exploration phase of the orange population, local optimization is performed to further improve the quality of the solution by simulating firefly interactions. The normalization of distance, an important part of the firefly perturbation algorithm [28], calculates the normalized distance $r_{i,j}$ between the current individual $X_{Orange}$ and the hypothetical optimal solution $X_{Best}$ for different sizes of the solution space as follows:

(28) $$r_{i,j}=\frac{\left\|norm\left(X_{Orange}-X_{Best}\right)\right\|}{d_{max}}$$

where $d_{max}=\left(ub-lb\right)\sqrt{dim}$.

Based on the exponential decay of the distance $r_{i,j}$, an attraction coefficient *β* is introduced to control the strength of the inter-individual interaction with the following equation:

(29) $$\beta =\beta_{0}e^{-\gamma \cdot r_{i,j}^{m}}$$

where $\beta_{0}=2,\;\gamma =1,\;m=2$ are preset parameters.

Its position update formula for the development phase is as follows:

(30) $$X_{new}=X_{Orange}+\beta \cdot \left(X_{Orange}-X_{Best}\right)+\alpha \left(rand\left(1,dim\right)-0.5\right)$$

In summary, the strategy of orange populations achieves a wide coverage of the search space in the exploration phase by optimizing the spiral search strategy and then uses the firefly perturbation strategy to finely optimize the potential high-quality solution regions in the exploitation phase, and this combination of strategies effectively balances the algorithm’s global exploration capability and local exploitation capability.

### 3.5. OOA and IOOA Basic Process

**Figure 4:**
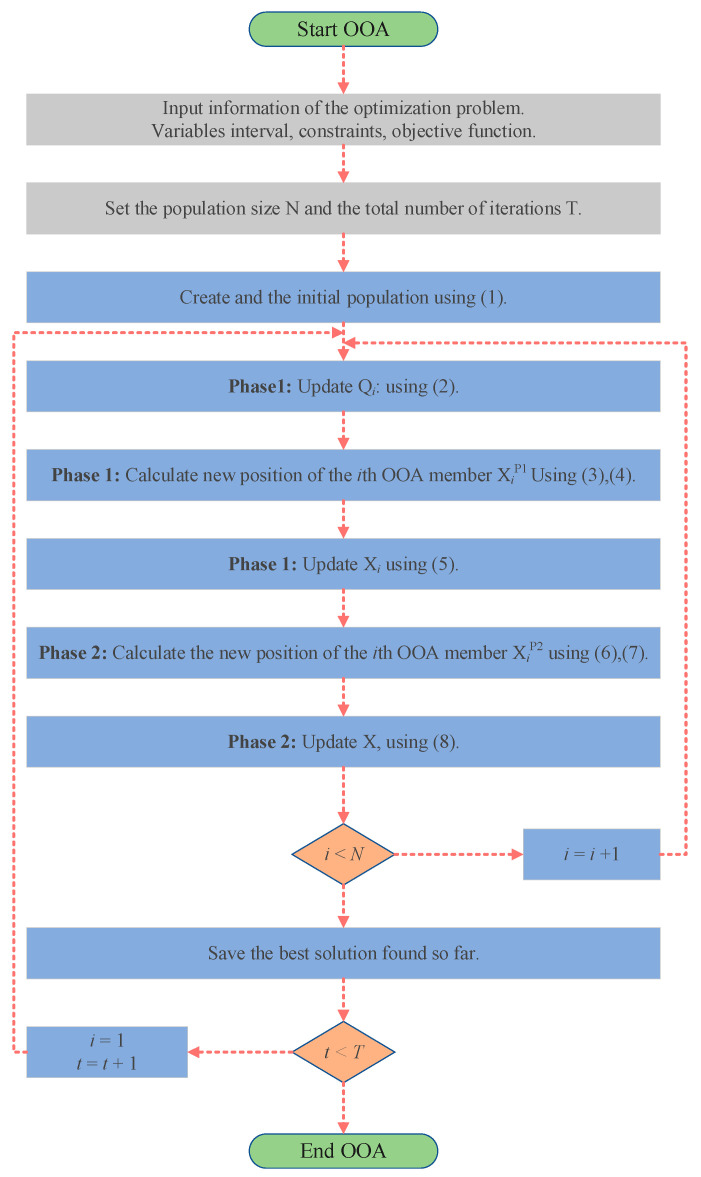
The flow chart of the OOA.

The pseudo-code of the OOA is shown in Algorithm 1 and Figure 4.
**Algorithm 1.** OOA steps can be summarized as follows.Input parameters: Osprey population size *N* and maximum number of iterations $iter_{max}$.Output parameters: best position $X_{Pos}$, best fitness value $X_{Best}$
**(1) Initialization phase**Initial population of fish eagles initialized using Equation (1).**(2) Algorithm flow:**The iteration begins:while (*t* < $iter_{max}$)  Initialize the array of fitness values.  for *i* = 1: N
**Phase 1: Position identification and hunting the fish**    Update fish positions set for the *i*th OOA member using Equation (2).    Determine the selected fish by the *i*th osprey at random.    Calculate new position of the *i*th OOA member based on the first phase of OOA using Equation (3).    Check the boundary conditions for the new position of OOA members using Equation (4).    Update the *i*th OOA member using Equation (5).**Phase 2: Carrying the fish to the suitable position**    Calculate new position of the *i*th OOA member based on the second phase of OOA using Equation (6).    Check the boundary conditions for the new position of OOA members using Equation (7).    Update the *i*th OOA member using Equation (8).  end  for *i* = 1: N
    Get the current new location;    If the new location is better than before, update it;  End for  *t* = *t* + *l*;  Get the current new location;  If the new location is better than before, update it;End while

**Figure 5:**
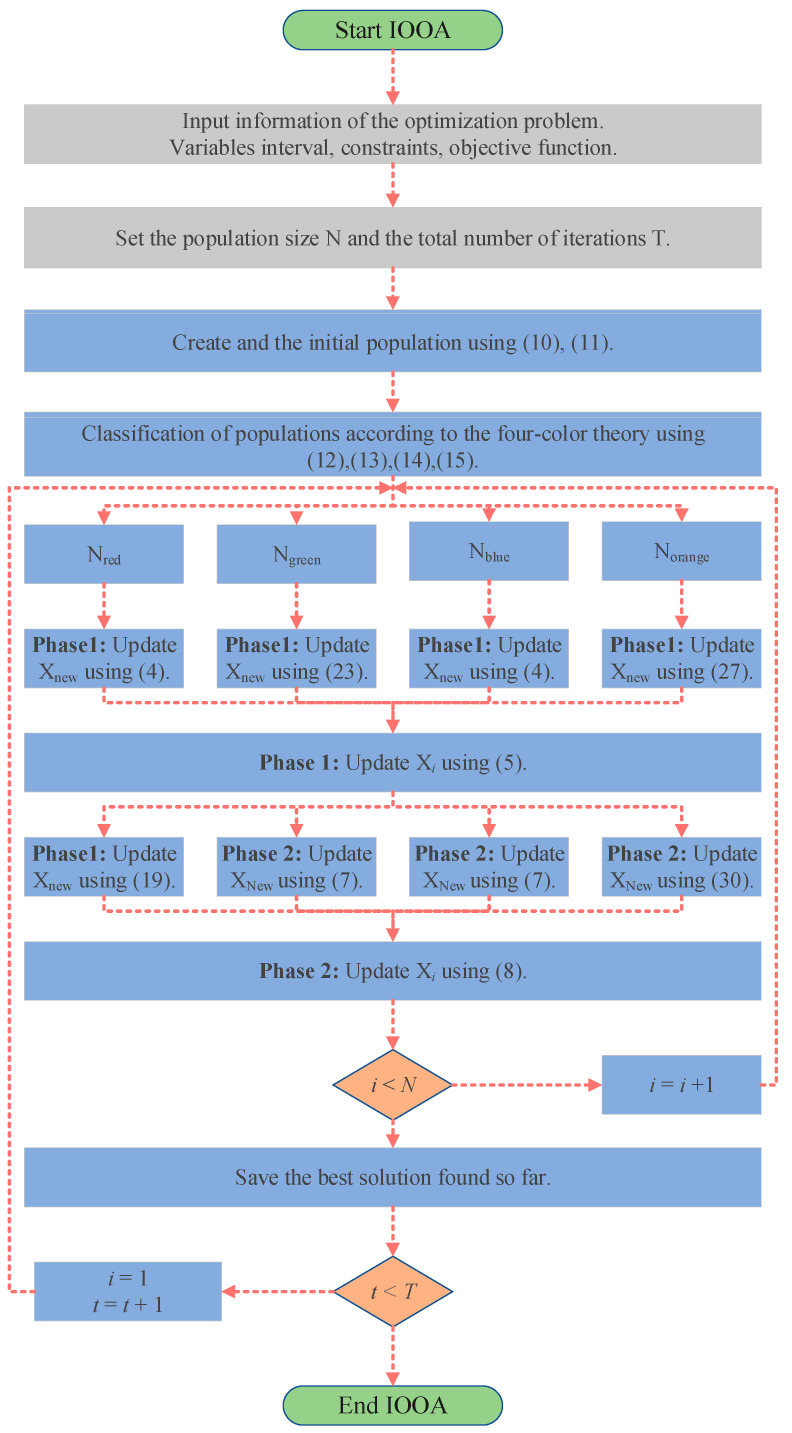
The flow chart of the IOOA.

IOOA is evolved from OOA, which enhances the performance of the algorithm through a series of strategic adjustments. First, in the initial stage, logistic chaos mapping and the good point set method are used to generate the initial population, and the population is divided into four different color groups according to the four-color theory as a way to improve the diversity of the population and balance the ability of global search and local exploitation. Then, a two-color complementary mechanism is introduced into the algorithm design, and a differentiated strategy is adopted for the populations of different color groups. For the blue population, the focus is on maintaining the original OOA exploration strategy to ensure the basic performance and stability of the algorithm. For the red population, a heuristic strategy based on the behavior of the Harris Hawk is introduced, which mimics the efficient search and localization of the Harris Hawk and improves the algorithm’s ability to avoid falling into local optimal solutions. The green population employs a strolling wandering strategy, which extends the search range by mimicking random wandering behavior while maintaining population diversity. Finally, the orange population adopts an optimized spiral search strategy to explore the solution space in depth and employs the firefly perturbation strategy to effectively perturb the local optimal solution, which facilitates the algorithm to jump out of the local optimum and continue to explore. The combination of these strategies enables IOOA to further improve the global search capability and the ability to avoid local optimums by inheriting the advantages of the original OOA. The flow charts of OOA and IOOA are shown in Figure 4 and Figure 5.

The pseudo-code of the OOA is shown in Algorithm 2.
**Algorithm 2.** IOOA steps can be summarized as follows.Input parameters: Osprey population size *N*, number of populations in the red group $N_{Red}$, number of populations in the green group $N_{Green}$, number of populations in the blue group $N_{Blue}$, number of populations in the orange group $N_{Orange}$, and maximum number of iterations $iter_{max}$.Output parameters: best position $X_{Pos}$, best fitness value $X_{Best}$
**(1) Initialization phase**The initial population of the fish eagle was initialized with the L-good point set using Equations (11) and (12), and then its initialized population was randomly grouped, and the red group of populations, the green group of populations, the blue group of populations, and the orange group of populations were selected according to Equations (13)–(16).**(2) Algorithm flow:**The iteration begins:while (*t* < $iter_{max}$)  Initialize the array of fitness values.  The exchange probability is calculated dynamically for each generation. According to equation (pt=max⁡p0×(1−δ)^t,pmin
  Setting the minimum exchange probability threshold to prevent the probability from being too small.  If $p_{t}\leq p_{min}$
    $p_{t}=p_{min}$  end  Blue stock location update  for *i* = 1: $N_{Blue}$
      Using Equation (5) update the blue population’s location;      Using Equation (8) update the blue population’s location;  end  Red stock location update  for *i* = 1: $N_{Red}$
      Using Equation (5) update the blue population’s location;      Using Equations (18) and (19) update the blue population’s location;  end  Green stock location update  for *i* = 1: $N_{Green}$
      Using Equation (23) update the blue population’s location;      Using Equation (8) update the blue population’s location;  end  Orange stock location update  for *i* = 1: $N_{Orange}$
      Using Equation (27) update the blue population’s location;      Using Equation (30) update the blue population’s location;  end  for *i* = 1: N
    Get the current new location;    If the new location is better than before, update it;  End for  *t* = *t* + *l*;End while

### 3.6. Complexity Analysis

In the standard OOA, let the osprey population size be *N* and the dimensionality of the solution space be *D*. OOA performs parameter initialization with a time complexity of *O*(*N* × *D*), individual fitness with *O*(*N*), and population complexity with *O*(*N* × *D*). Since these operations are performed for each individual of each population and involve fitness function calls (assuming *O*(*D*) time complexity for fobj), the total time complexity is *O*(*M* × *N* × *D*). So, the overall complexity of OOA is as follows:

(31) O(OOA)=O(N×D)+O(N)+O(M×N×D)=O(M×N×D)

The IOOA algorithm first generates an *N* × *D* random matrix using a logistic mapping in the initialization phase, and next, two loops are performed: an outer loop *i* from 1 to *N* and an inner loop *j* from 2 to *D*. The logistic mapping is computed each time the inner loop is performed, which is a constant time operation. Therefore, the time complexity of this part is *O*(*N* × *D*).

The good point set strategy mainly uses a prime-based operation, which has a time complexity of *O*(*N* × *D*) because it involves computing an expression for each dimension and copying it to all individuals, which is *O*(*N* × *D*) overall.

Combined with the above analysis, the most time-consuming parts of the initialization process are the computation of the logistic mapping, the combination operation with the good point set strategy, and the generation of random permutations. Among them, the generation of random permutations is *O*(*N* × *D*), and all other major steps are *O*(*N* × *D*). Therefore, considering the worst-case dominant term, the time complexity of the whole initialization function is roughly *O*(*N* × *D*). During the operation of IOOA, there is mainly the exchange probability calculation, which is a constant time complexity *O*(1). The individual exchange part between populations is performed separately for red and green populations and blue and orange populations. The time complexity of the exchange operation for each color population is *O*(*N* × $P_{t}$), but the actual number of operations decreases as $P_{t}$ decays over time. Overall, the time complexity of this part is close to O(N) if the average exchange probability is considered. The time complexity calculations for each color group population separately are O($N_{Red}$ × *D*), O($N_{Green}$ × D), O($N_{Blue}$ × D), and O($N_{Orange}$ × D), respectively. Different update rules are applied to each population, including the calculation of new positions and fitness values. Since these operations are performed for each individual of each population and involve fitness function calls (assuming *O*(*D*) time complexity for fobj and *O*(*M* × ($N_{Red}$ + $N_{Green}$+ $N_{Blue}$ + $N_{Orange}$) × *D*) for the total time complexity). Recording the optimal adaptation per generation is a typical linear operation with a time complexity of *O*(*M*). The total time complexity of this IOOA is as follows:

(32) $$\begin{array}{c} O\left(IOOA\right)=O\left(N\times D\right)+O\left(1\right)+O\left(N\right)+O\left(M\times \left(N_{Red}+N_{Green}+N_{Blue}+N_{Orange}\right)\times D\right)\\ =O\left(M\times N\times D\right) \end{array}$$

In summary, IOOA does not show a significant increase in time complexity compared to OOA.

## 4. Experimental Simulation and Result Analysis

In this chapter, a simulation study will be conducted to evaluate the effectiveness of IOOA in optimization. The experiments were conducted on MATLAB R2021b with an 11th-generation Intel Core i7 processor, 16 GB of dual-channel RAM, and a 512 GB SSD. The graphics card is a GeForce RTX 3050Ti.

### 4.1. Selection of Benchmark Function and Experimental Setup

This paper evaluates the performance of the IOOA algorithm in handling various objective functions by using 30 test functions, including 8 benchmark functions, the CEC2020 test set, and the CEC2022 test set [29]. The study compares IOOA with ROA, SFO, CDO, BA, GSA, WWPA, OOA, and two improved SOOA and ADSOOA to assess the quality of the best solution provided by IOOA. The control parameters of all the algorithms are set according to the recommendations of the algorithm proposers and are described in detail in Table 1.

The above is a selection of the static parameters of the chosen algorithm, and the following will focus on the three main dynamic parameters in IOOA: *P*, *a*, and *A*.

**Figure 6:**
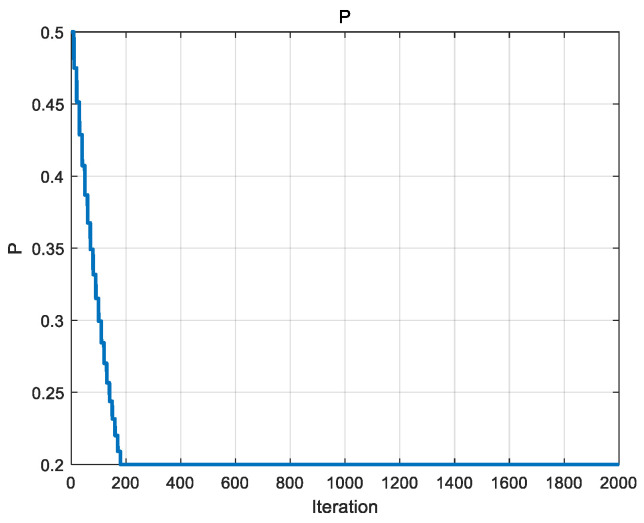
Variation in *P*-value with number of iterations.

*P* is a parameter in the exploration phase used to control the probability of individual exchange between populations. The initial value of *P* is set to 0.5, and this parameter decreases over time, allowing the algorithm to move from extensive exploration in the early stages to refined exploitation in the later stages. As the number of iterations proceeds to a certain level, the value of *P* will be maintained to minimize the exchange probability, and in this paper, the minimum exchange probability threshold *p_min_* is set to 0.2. The variation in *P* with the number of iterations is shown in Figure 6.

**Figure 7:**
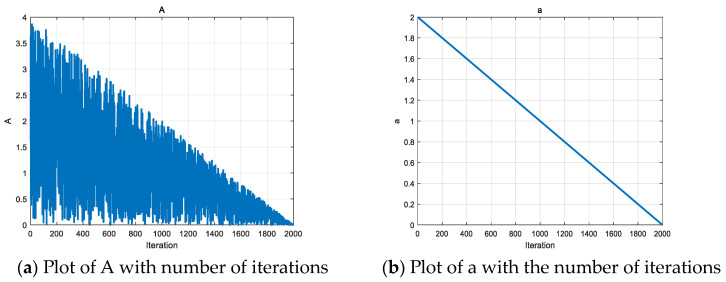
Variation in *A* and *a* with number of iterations.

*A* is the deflation factor in the black population, which is used to control the search radius in the spiral search strategy. *a* is a parameter that decays linearly with the number of iterations, which can control the search radius of *A* well with the increase in the number of iterations, so *A* will decrease linearly with the increase in the number of iterations, which will gradually narrow down the search range and improve the development of the algorithm. Refer to Equations (25) and (27) for details. At the beginning of the iteration, *A* is larger, which is favorable for the algorithm to conduct a comprehensive search of the problem space; as the number of iterations increases, A gradually decreases, which helps the algorithm to transition from the exploration stage to the development stage, and thus more accurately approximate the global optimal solution. The variation in *A* and *a* with the number of iterations is shown in Figure 7.

In the experimental design, the performance test of the algorithm is gradually transitioned from the benchmark test set to the CEC2020 test set and then to the CEC2022 test set. Its details are shown in Table 2, Table 3 and Table 4 in turn. This gradual transition aims to evaluate the performance of the algorithms on different test sets in depth and to provide a more accurate basis for performance evaluation. The number of populations N, the maximum number of iterations T, and the dimension Dim of each algorithm on different test sets in the experiment are shown in Table 5. To reduce the chance of the experiment and increase the persuasiveness of the experimental results, each algorithm is run independently 10 times on each benchmark test function. To analyze the performance of the algorithms, convergence analysis, stability analysis, and nonparametric tests are performed. Convergence analysis includes analyzing performance using convergence graphs and convergence tables, where the convergence table contains four evaluation functions such as best, average, standard deviation (Std), and number of iterations. These analyses help to understand the convergence speed and stability of each algorithm and its performance in solving problems. Stability analysis was performed using the ANOVA test [30] for variance and box line plots to indicate the stability of the algorithms. Nonparametric tests such as the Wilcoxon signed rank test [31] and the Friedman rank sum test [32] were also used to delve into the statistical significance of the differences in the performance of the algorithms on different problem instances.

The experiments and analyses in this study aim to comprehensively evaluate the performance of the IOOA algorithm in dealing with various objective functions and provide strong comparative and statistical evidence for further research and applications.

### 4.2. Convergence Analysis

#### 4.2.1. Convergence Analysis of IOOA on Benchmark Test Set

From the data in Table 6, it can be observed that the IOOA shows excellent performance on several test functions. Specifically, on the test functions F1 to F3, F5, F6, and F8, the minimum, mean, and standard deviation obtained by the IOOA algorithm are the smallest among all the algorithms, which fully demonstrates that its optimization on these test functions is the best. Meanwhile, in the F3 test function, apart from the IOOA algorithm, the ROA, GSA, WWPA, OOA, SOOA, and ADSOOA algorithms also found optimal values. Although in the F4 test function, the IOOA algorithm does not have as good a minimum, mean, and standard deviation as the ADSOOA algorithm, the answers found by the IOOA algorithm are extremely close to the results of the ADSOOA algorithm, which indicates that both algorithms perform quite well on this test function. In the F7 test function, although the mean and standard deviation of the IOOA algorithm are not the smallest among all the algorithms, the minimum value found by it is still the smallest among all the algorithms, which also highlights the significant advantage of the IOOA algorithm in finding the optimal solution. In summary, the IOOA algorithm shows excellent optimization performance on most of the tested functions.

**Figure 8:**
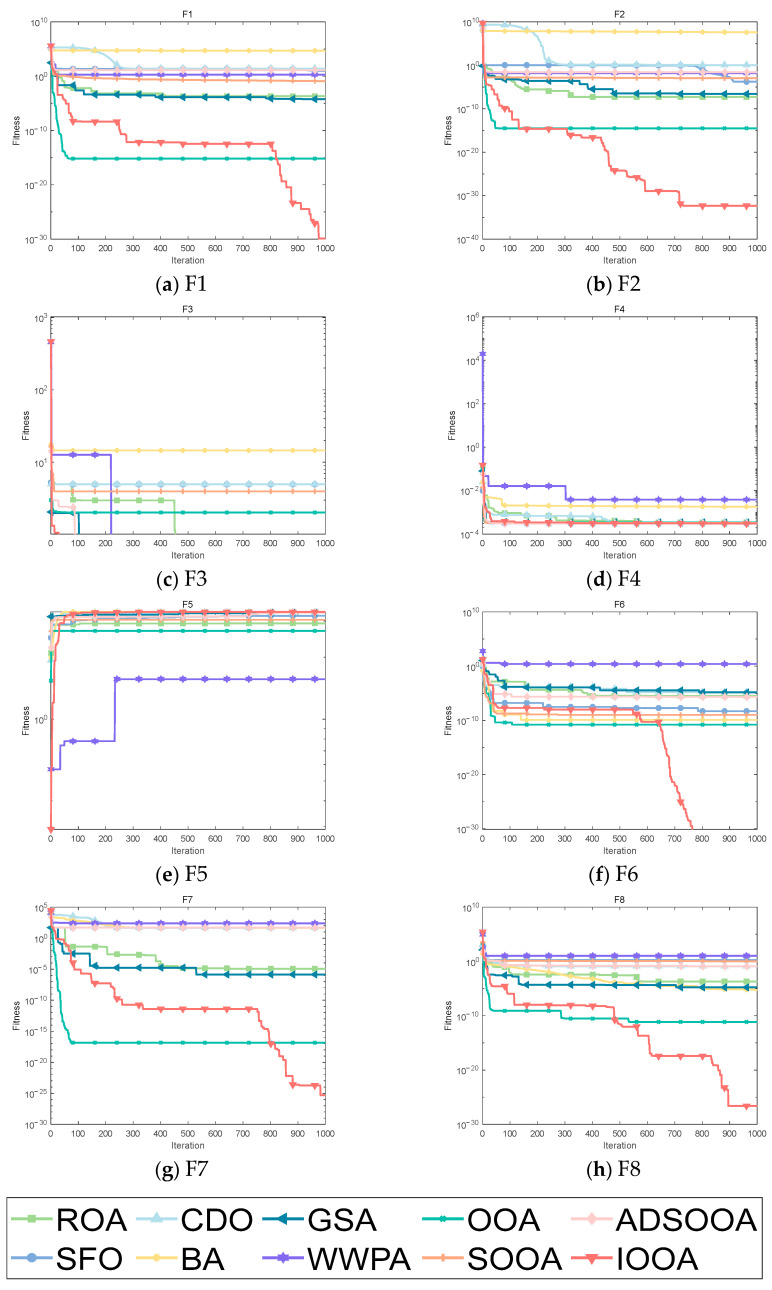
Convergence analysis on benchmark functions.

It can be clearly observed from Figure 8 that IOOA shows excellent convergence accuracy and accelerated convergence in all test functions. Especially on the test functions F1, F2, and F6 to F8, IOOA has a clear advantage in convergence accuracy compared to other algorithms. In addition, on the three test functions from F3 to F5, although the convergence accuracies achieved by multiple algorithms do not differ much, IOOA also shows good performance. Especially noteworthy is that in the F3 test function, IOOA’s convergence speed is significantly faster than the other algorithms, which further reflects its optimization ability in this test function. In summary, IOOA shows excellent convergence performance and accuracy on several test functions.

#### 4.2.2. Convergence Analysis of IOOA on CEC2020 Test Set

From the data in Table 7, it can be observed that the IOOA algorithm exhibits excellent performance on several test functions. Specifically, on four test functions, F9, F12, F16, and F18, IOOA obtained the smallest minimum, mean, and standard deviation, indicating that it optimized best on these four functions. For the three test functions F10, F11, F14, and F15, although the standard deviation of IOOA is not the smallest, its minimum and mean values are at the optimal level, which further proves the optimization ability of IOOA. In addition, when comparing IOOA with CDO in test function F13 and IOOA with ADSOOA in test function F17, although the mean and standard deviation of IOOA are not the smallest, the minimum value it finds is still the smallest among all the algorithms, which likewise demonstrates the advantage of IOOA in finding the optimal solution. In summary, the IOOA algorithm shows excellent optimization performance on most of the tested functions.

**Figure 9:**
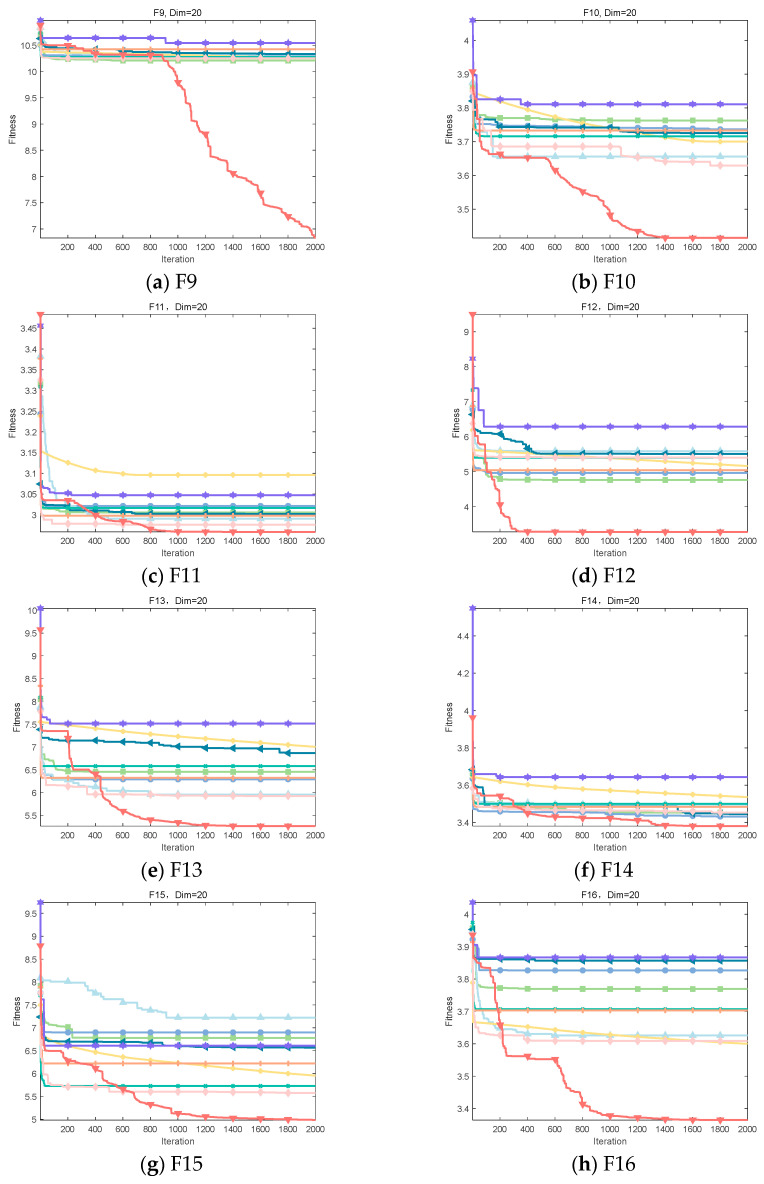

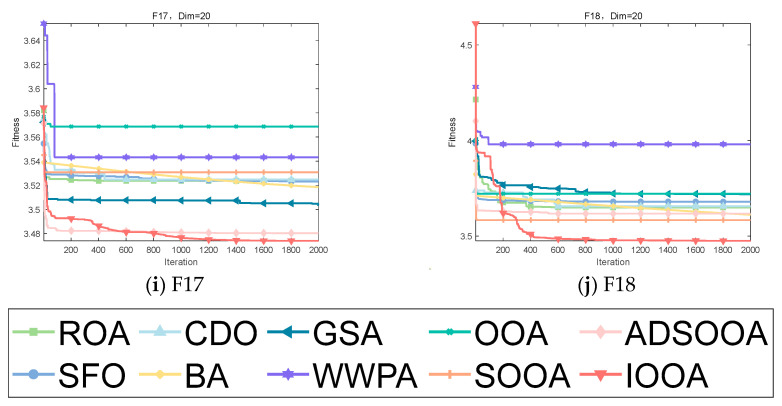
Convergence analysis on CEC2020.

It is clear from Figure 9 that IOOA demonstrates excellent convergence accuracy as well as the ability to accelerate convergence in all the tested functions. In particular, for the functions F9, F10, F12, F13, F15, and F16, the convergence accuracy of IOOA has a clear advantage over other algorithms. In addition, on the test functions F11 and F17, ADSOOA follows IOOA and also shows good performance.

#### 4.2.3. Convergence Analysis of IOOA on CEC2022 Test Set

As can be seen from Table 8, the IOOA algorithm demonstrates excellent performance on several test functions, especially on the four functions F19, F24, F27, and F29, which are particularly optimized. IOOA not only achieves the minimum on these four functions but also outperforms the other algorithms in terms of the mean and stability of the results (measured in terms of standard deviation), which highlights its excellent performance on these tests. For the F20, F26, and F28 test functions, although IOOA is not optimal in terms of stability (standard deviation) of the results, it is able to consistently achieve and maintain the lowest minimum and mean values, which further validates the power and stability of the IOOA algorithm in complex optimization problems. In the F22 and F23 test functions, although IOOA is not optimal in terms of the mean and standard deviation metrics, it successfully mines the smallest minima among all participating algorithms, which again emphasizes the unique advantages of IOOA in deep searching and discovering globally optimal solutions. In summary, the IOOA algorithm generally demonstrated excellent optimization performance on a wide set of test functions, not only achieving the best overall performance on multiple functions but also highlighting its efficiency and accuracy in finding optimal solutions on specific functions. These results fully demonstrate the potential and value of the IOOA algorithm in the field of optimization.

**Figure 10:**
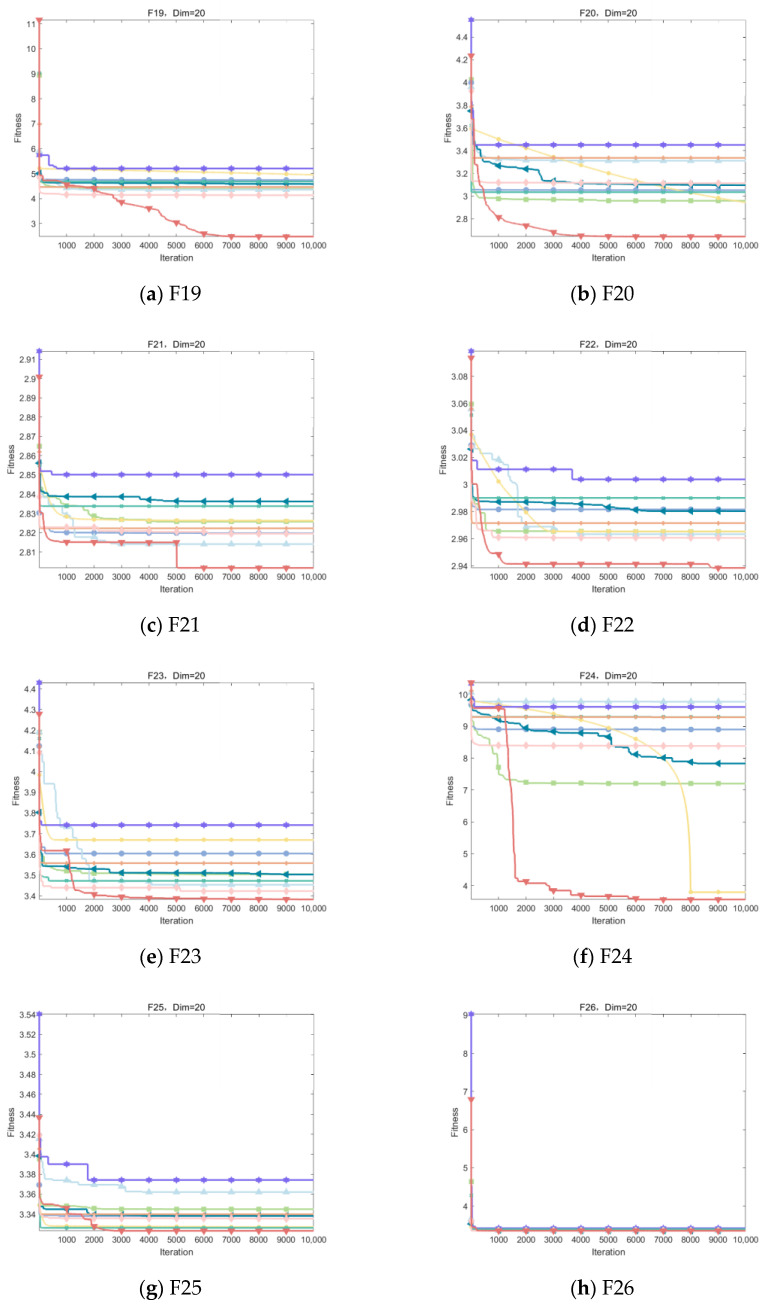

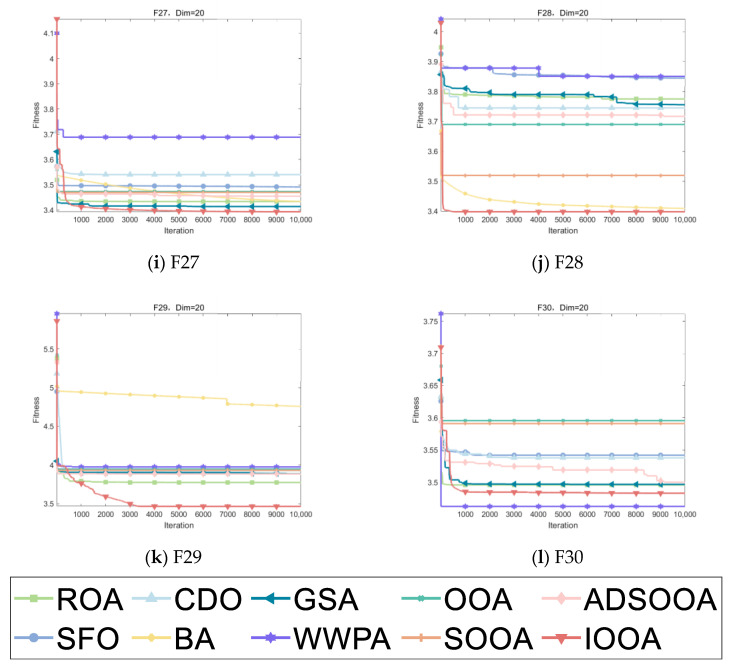
Convergence analysis on CEC2022.

Figure 10 visualizes the excellent performance of IOOA in all the tested functions, not only in its high convergence accuracy but also in its remarkable ability to accelerate convergence. In particular, on the functions F19 to F22, F24, and F29, the convergence accuracy of IOOA far exceeds that of other algorithms, showing a clear advantage. It is worth noting that on the F23 function, although IOOA still takes the lead, ADSOOA follows closely and also performs well, showing its unique optimization ability. However, on these functions from F25 to F27, IOOA’s performance is comparable to that of the other algorithms, indicating that different algorithms have their own adaptability to different types of problems. In summary, IOOA shows excellent optimization performance on most of the tested functions.

### 4.3. Stability Analysis

A powerful data visualization tool, box-and-line charts excel at demonstrating the distributional properties of continuous data sets, especially concentrated trends, dispersion, and possible extreme data points. The chart is built on top of several key statistics: lower quartile, upper quartile, median, and a body consisting of boxes and whiskers, with occasional outliers labeled. The box encloses 50% of the overall data, bounded by the lower and upper quartiles, while the median falls inside the box, reflecting the center of the data. The whiskers, on the other hand, extend outward to the regular boundaries of the data and are generally defined within 1.5 times the expansion of the interquartile spacing, with points beyond this boundary considered outliers.

Box-and-line plots are particularly important when evaluating algorithm performance, as they visualize the similarities and differences in how algorithms perform when faced with the same task or in different contexts. The compactness of the box directly reflects the concentration of data and the consistency of the algorithm’s results, while lower box positions imply the algorithm’s ability to find high-quality solutions. Comparing the boxplots of multiple algorithms, algorithms with more robust performance on specific tasks, higher solution accuracy, and lower sensitivity to external interference can be quickly identified, providing a strong visual basis for algorithm selection and tuning.

#### 4.3.1. IOOA Stability Analysis on Benchmark Test Sets

**Figure 11:**
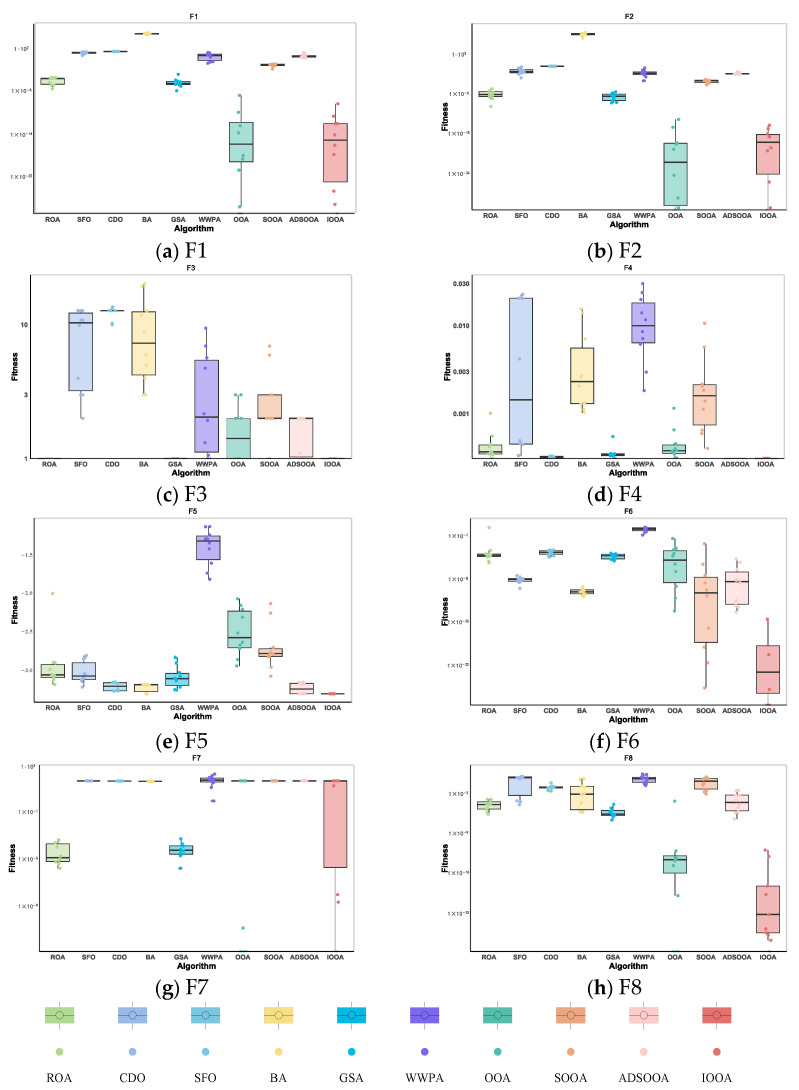
Stability analysis on benchmark functions.

As can be observed in Figure 11, IOOA shows its excellent performance stability and advantages through box-and-line plots on this test function. Specifically, for the test functions F6 and F8, IOOA’s boxplot shows a lower median value, which indicates that its performance is relatively better on these two functions. In addition, for the test functions F3, F4, and F5, the boxplots of IOOA show excellent stability, indicating that its performance fluctuates less on these functions. On the other hand, for the test functions F1, F2, and F7, IOOA is able to find even smaller values, further demonstrating its ability to optimize these functions. In summary, IOOA shows excellent performance and stability on several test functions.

#### 4.3.2. IOOA Stability Analysis on CEC2020 Test Sets

**Figure 12:**
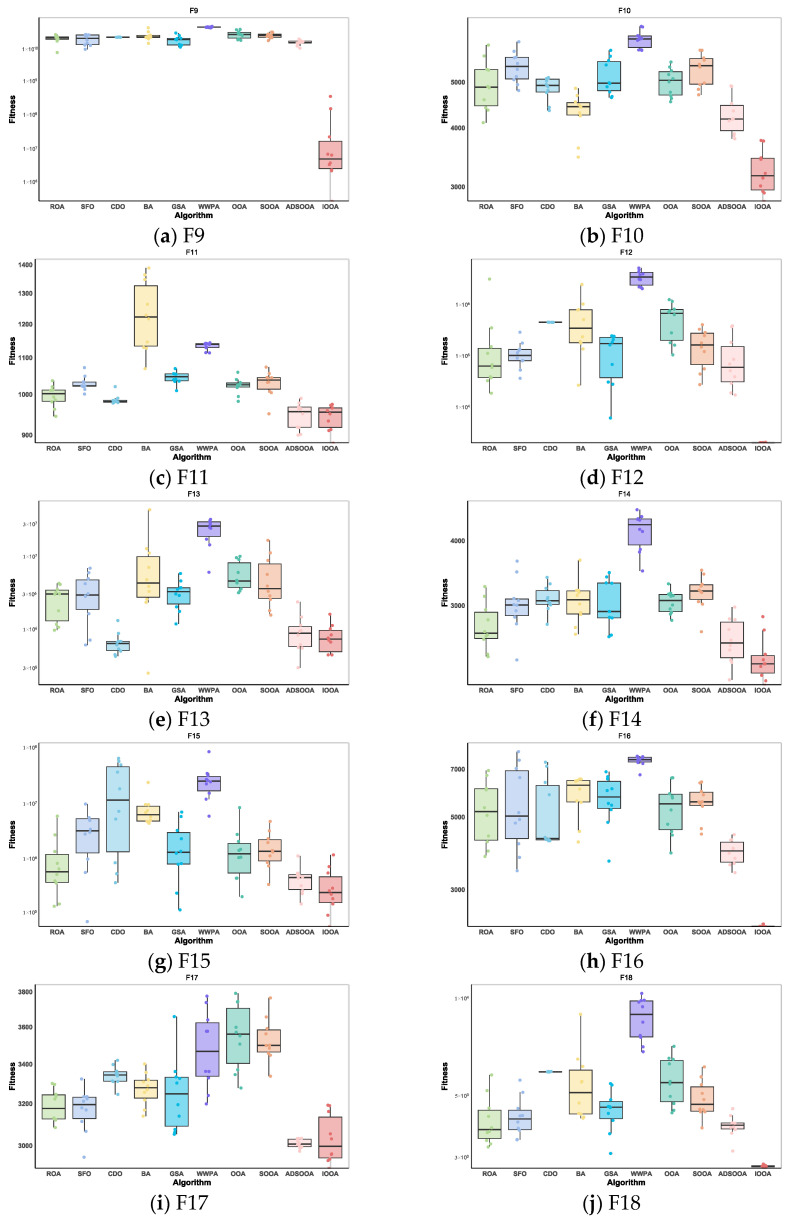

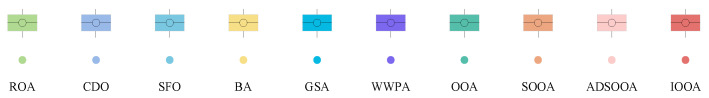
Stability analysis on CEC2020.

As can be observed from Figure 12, IOOA shows excellent performance stability and benefits through box-and-line plots for the set of test functions of CEC2020. For most of the test functions, the boxplots of IOOA show lower median values, especially more prominent for the functions F9, F10, F12, F14, F16, and F18. In addition to this, IOOA shows excellent stability for the functions F9, F12, F16, and F18. This intuitively shows that the algorithm is able to produce the smallest variance during the optimization of these types of functions, which means that it is able to provide the most stable solutions.

#### 4.3.3. IOOA Stability Analysis on CEC2022 Test Sets

**Figure 13:**
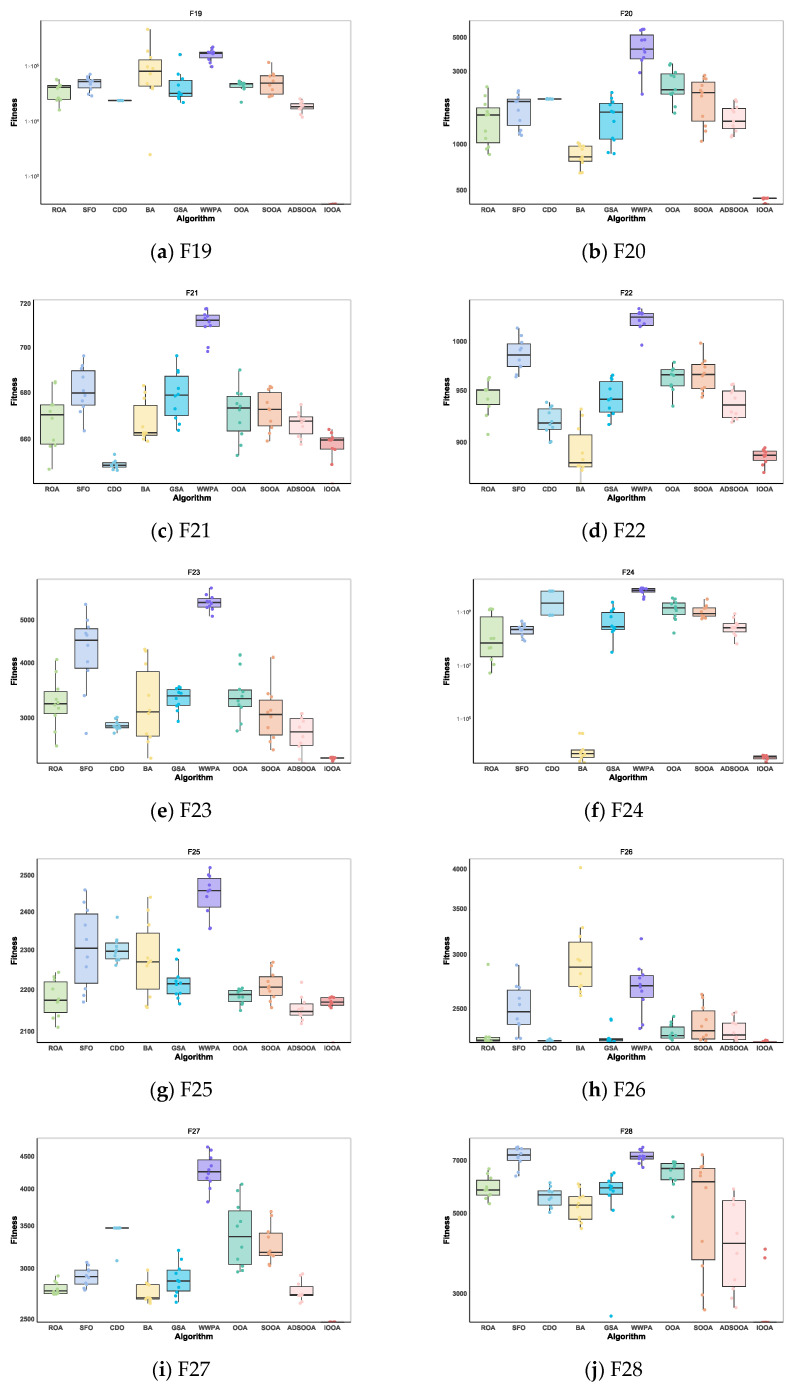

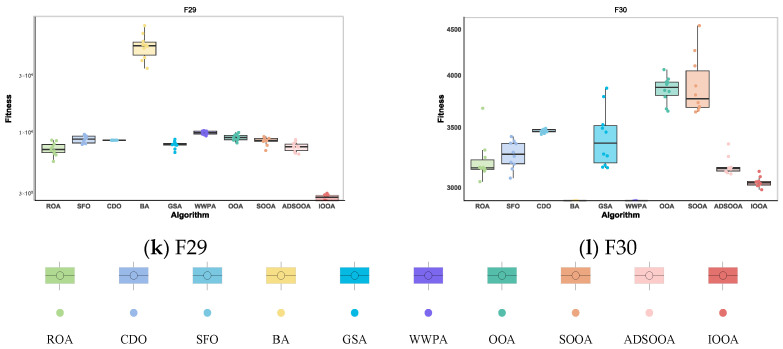
Stability analysis on CEC2022.

In the presentation in Figure 13, IOOA’s performance on the CEC2022 test function set is particularly impressive, with extremely significant performance stability and advantages reflected in its box-and-line plots. Specifically, IOOA maintains low median values on most of the test functions, and this advantage is particularly strong for functions F19 through F24, F26, and F30, showing the algorithm’s superior performance on these functions. For the functions F19, F20, F26, and F30, IOOA not only performs well but also shows great stability. The extremely small variance range in the boxplots visually demonstrates this, implying that when optimizing these functions, IOOA is able to generate highly consistent found optimal solutions, reducing the volatility and uncertainty of the results. In summary, IOOA’s performance on the CEC2022 test function set proves its excellent performance stability and significant advantages on specific function types, providing a powerful tool for solving complex optimization problems.

### 4.4. Nonparametric Test

Nonparametric tests play an indispensable role in the statistical evaluation of algorithm performance, especially when dealing with situations where the data distribution is unknown or deviates from normality. Wilcoxon rank sum test and Friedman’s test, as two widely recognized nonparametric analytical tools, are particularly useful in the comparative study of algorithm performance, which not only overcame the stringent requirements of the distribution pattern of the data but also deeply explored the statistical significance of performance differences between algorithms.

Specifically, the Wilcoxon signed rank test is good at analyzing paired data or single-sample situations and reveals the significance of the internal differences of algorithms by comparing their performance changes in different conditions or points in time. On the other hand, Friedman’s test, from a broader perspective, focuses on the overall performance distribution of all algorithms under multiple levels (e.g., multiple test functions) and detects whether there is a significant difference between algorithms by calculating the average ranking of each algorithm, which is especially adapted to experimental data of round-robin or paired designs.

#### 4.4.1. IOOA Nonparametric Analysis on Benchmark Test Sets

As can be seen from Table 9, IOOA excels in performance. It significantly outperforms SFO, WWPA, and SOOA on all eight test functions; significantly outperforms CDO, BA, and ADSOOA on seven test functions when comparing with CDO, BA, and ADSOOA; significantly outperforms ROA and GSA on six test functions when comparing with ROA and GSA, but there is no significant difference in F3 and F7; and significantly outperforms OOA on half of the test functions when comparing with OOA and has no significant difference with OOA on the other half of the no significant difference with OOA on the other half of the test functions. In summary, IOOA demonstrates excellent performance on several test functions and shows significant advantages over multiple algorithms.

**Figure 14:**
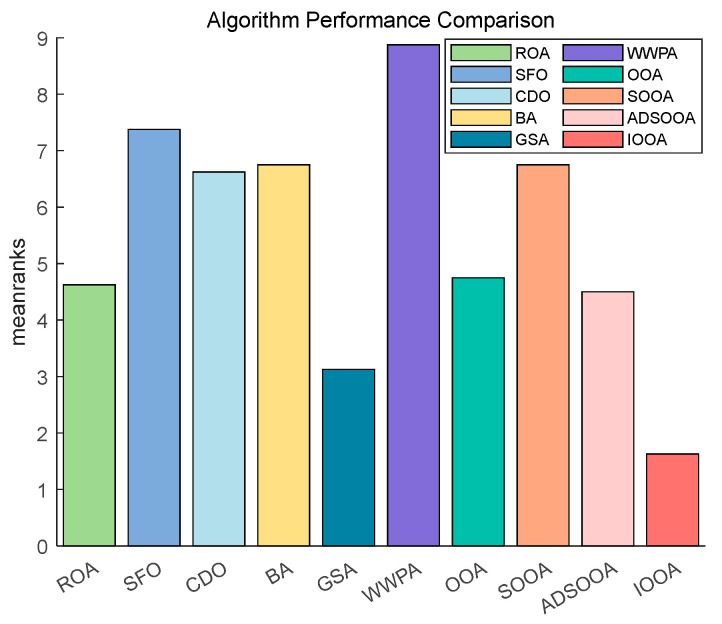
The Friedman test results on benchmark functions.

Figure 14 shows that IOOA has the highest ranking on this test function, indicating the best performance followed by GSA. OOA and ADSOOA are third and fourth. ROA, CDO, BA, SOOA, SFO, and WWPA, the average scores of these algorithms are in increasing order. This indicates that IOOA shows a significant advantage in this test function.

#### 4.4.2. IOOA Nonparametric Analysis on CEC2020 Test Sets

As can be seen from Table 10, IOOA is significantly better than ROA on 9 test functions and is not significantly different from ROA on F15.IOOA is significantly better than SFO, BA, GSA, WWPA, and OOA on all 10 test functions. IOOA is significantly better than CDO on 9 test functions and is not significantly different from CDO on F13.IOOA is significantly better than BA on 9 test functions and is not significantly different from CDO on F13, significantly better than BA, and is not significantly different from BA on F11. IOOA is significantly better than SOOA on 9 test functions and is not significantly different from SOOA on F15. IOOA is significantly better than ADSOOA on 5 test functions and is not significantly different from ADSOOA on F5. In summary, IOOA significantly outperforms all other algorithms except ADSOOA on most of the test functions.

**Figure 15:**
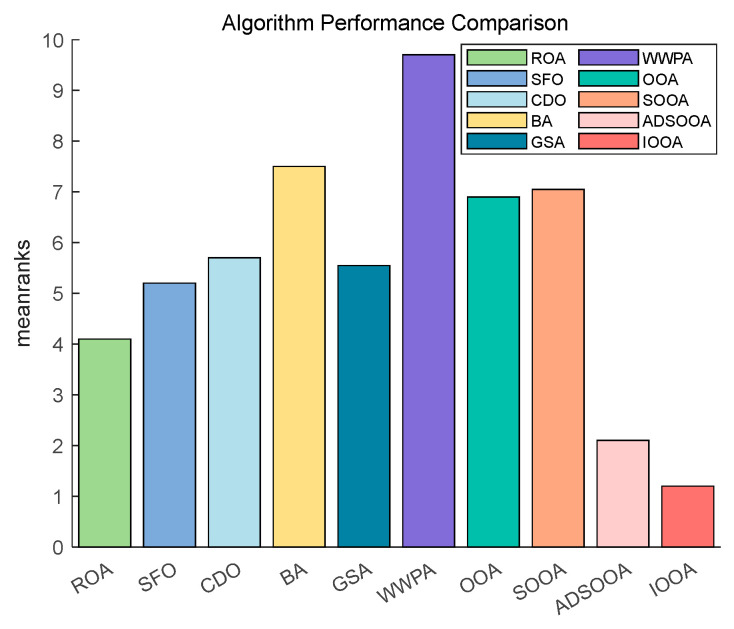
The Friedman test results on CEC2020.

Figure 15 shows that IOOA is the top performer in all functions. IOOA and ADSOOA are the best performers, ranked first and second, respectively. ROA, SFO, GSA, and CDO are close to each other and ranked in the middle of the list. BA and WWPA are worse performers, ranked ninth and tenth, respectively.

#### 4.4.3. IOOA Nonparametric Analysis on CEC2022 Test Sets

As can be seen from Table 11, IOOA significantly differs in performance compared to the other algorithms in most cases. As can be seen from the +/=/− columns, IOOA significantly outperforms the other algorithms in the vast majority of comparisons and is only significantly inferior to the other algorithms in very few cases. In particular, in the comparisons with SFO, GSA, and SOOA, IOOA significantly outperforms these algorithms on all functions (F19 through F30). For some functions (e.g., F21), there is no significant difference in performance between IOOA and some algorithms (e.g., ROA), and it is even significantly inferior to CDO in some cases (vs. CDO), which may indicate a limitation of IOOA for some specific problems or function types. For other functions (e.g., F25), there is no significant difference in performance between IOOA and multiple algorithms (e.g., ROA, OOA, ADSOOA), which may be related to the complexity of the problem or the characteristics of the algorithm. When compared to BA, on F22 and F24, there is no significant difference, and it is inferior to BA on F30. In conclusion, IOOA shows a significant performance advantage in most of the comparisons with multiple algorithms on CEC2022.

**Figure 16:**
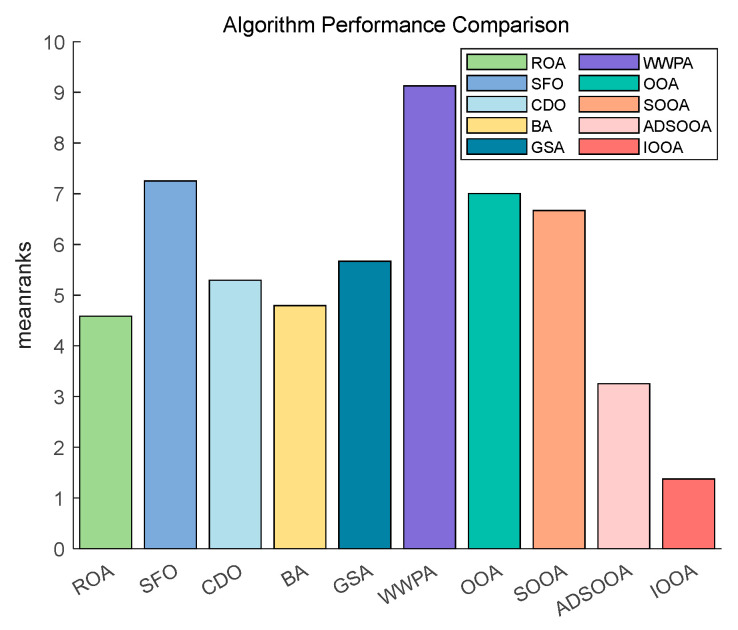
The Friedman test results on CEC2022.

Figure 16 clearly demonstrates the excellent performance of IOOA on all the tested functions, placing it firmly in the first place and highlighting its best performance, followed by ADSOOA, which performs well, and BA, ROA, CDO, and GSA, which perform equally well and are located in the middle of the ranking. In contrast, SOOA, OOA, SFO, and WWPA are gradually moving down the rankings. This result fully proves that IOOA has a significant performance advantage on the current set of test functions.

## 5. Engineering Applications of the IOOA

In this chapter, the potential of IOOA in solving typical engineering design challenges is explored, and its powerful optimization capabilities and practical benefits are demonstrated through three examples. These three cases are the design of tension and compression springs, the optimization of a welded beam problem, and the design and optimization of a gear transmission system, all of which are common and complex problems in the engineering field and place high demands on the performance of the algorithms. The breadth and depth of the application of IOOA in real engineering problems are comprehensively examined. First, in the design of tension and compression springs, faced with the comprehensive consideration of material utilization, strength, and elasticity properties, IOOA successfully finds the optimal design solution to minimize the spring mass under the given constraints. Secondly, for the welded beam problem, IOOA demonstrated its excellent ability to optimize the structural parameters under multiple constraints, effectively reducing the manufacturing cost while ensuring the safety and reliability of the structure. Finally, in gear train optimization, IOOA further proves its efficiency and accuracy in improving the efficiency of mechanical systems by minimizing the transmission ratio.

### 5.1. Extension/Compression Spring Design

**Figure 17:**
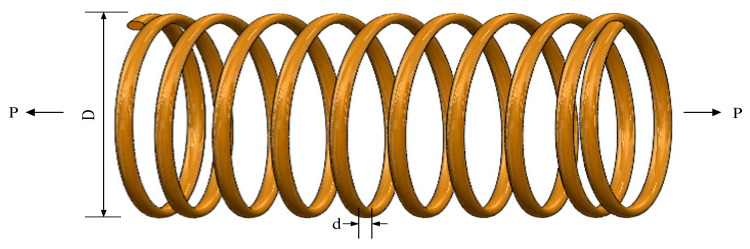
An illustration of the schematic structural diagram of a tension/compression spring.

When performing an optimized configuration of an extension/compression spring, the core design elements involve the spring wire diameter *d* (denoted as ($x_{1}$)), the average diameter of the spring coils (*D*) (labeled ($x_{2}$)) and the number of coils (*P*) (denoted ($x_{3}$)). The design aims to minimize the overall mass of the spring while ensuring that key performance indicators such as deflection, shear stress, and vibration frequency meet preset criteria. The structural layout of the spring is shown graphically in Figure 17.

The problem of tension spring design is modeled as the following mathematical optimization formulation:

(33) $$minf\left(x\right)=x_{1}^{2}x_{2}x_{3}+2x_{1}^{2}x_{2}$$

(34) $$s.t.\left\{\begin{array}{c} g_{1}(x)=1-\frac{x_{2}^{3}x_{3}}{71785x_{2}^{4}}\leq 0\\ g_{2}(x)=\frac{4x_{2}^{2}-x_{1}x_{2}}{12566(x_{1}^{3}x_{2}-x_{1}^{4})}+\frac{1}{5108x_{1}^{2}}-1\leq 0\\ g_{3}(x)=1-\frac{140.45x_{1}}{x_{2}^{2}x_{3}}\leq 0\\ g_{4}(x)=\frac{x_{1}+x_{2}}{1.5}-1\leq 0\\ 0.05\leq x_{1}\leq 2,\;\;0.25\leq x_{2}\leq 1.3\\ 2\leq x_{3}\leq 15 \end{array}\right.$$

**Figure 18:**
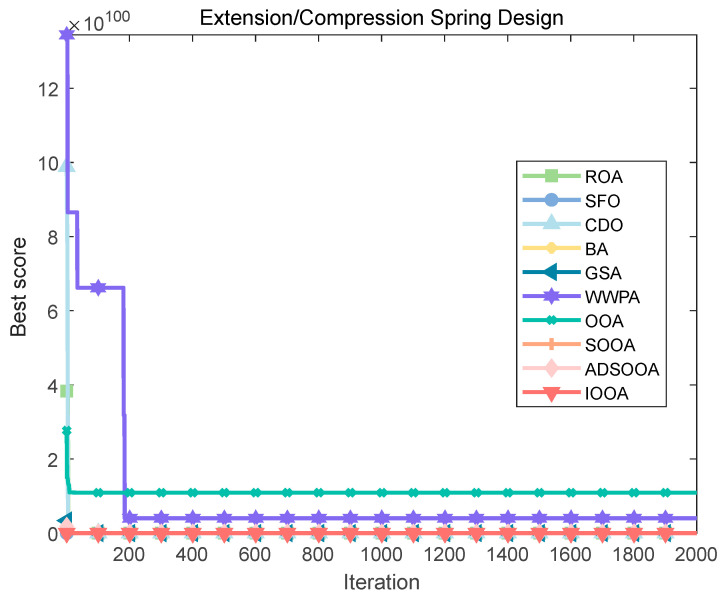
Iterative curves of various algorithms for a tension/compression spring.

The IOOA algorithm is employed to address the above spring design challenges, and its performance is compared with seven other algorithms. The comparison results are summarized in Table 12 and Figure 18, which clearly show that the IOOA algorithm is finding the best set of design parameters $\left(x_{1},x_{2},x_{3}\right)$. and the lowest quality *f*min(*X*) with a specific value of 1.27 × 10^−02^. This remarkable result validates the efficiency and leading edge of the IOOA algorithm in solving the design challenges of tension springs.

### 5.2. Optimized Design Problems for Welded Beams

**Figure 19:**
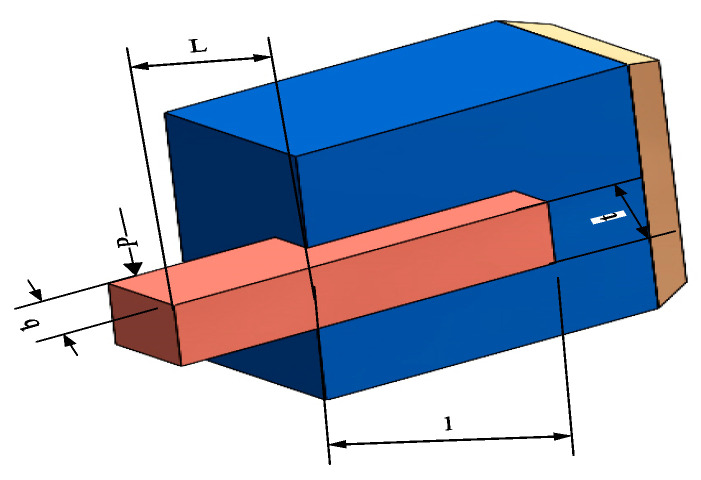
Schematic diagram of three-rod truss structure.

The optimal design challenge for welded beams is a complex problem that combines structural mechanics and mathematical optimization, aiming to ensure that a set of engineering constraints are satisfied by tuning the design parameters *X* = (*h*, *l*, *t*, *b*), i.e., the height, length, thickness, and width of the beams while ensuring that the premise of minimizing the manufacturing cost. Specifically, this process considers the shear stress *τ*, the bending stress *σ*, the bending load *Pc* imposed on the beam, the maximum deflection δ at the end of the beam, and the geometric boundary constraints, all of which need to be controlled to stay within the safety margins. The model is shown in Figure 19.

The design objective function is expressed as minimizing cost:

(35) $$minf\left(x\right)=1.10471x_{1}^{2}x_{2}+0.04811x_{3}x_{4}\left(14.0+x_{2}\right)$$

(36) $$s.t.\left\{\begin{array}{c} g_{1}\left(X\right)=\tau \left(x\right)-\tau_{max}\leq 0\\ g_{2}\left(X\right)=\sigma \left(x\right)-\sigma_{max}\leq 0\\ g_{3}\left(X\right)=\delta \left(x\right)-\delta_{max}\leq 0\\ g_{4}\left(X\right)=x_{1}-x_{4}\leq 0\\ g_{5}\left(X\right)=P-P_{c}\left(x\right)\leq 0\\ g_{6}\left(X\right)=0.125-x_{1}\leq 0\\ g_{7}\left(X\right)=1.10471x_{1}^{2}+0.04811x_{3}x_{4}\left(14.0+x_{2}\right)-5.0 \end{array}\right.$$

where

(37) $$\left\{\begin{array}{c} \tau \left(x\right)=\sqrt{{\left(\tau^{\prime }\right)}^{2}+2\tau^{\prime }\tau^{\prime \prime }\frac{x_{2}}{2R}+{\left(\tau^{\prime }\right)}^{2}}\\ \tau^{\prime }=\frac{p}{\sqrt{2x_{1}x_{2}}}\\ \tau^{\prime \prime }=\frac{MR}{J}\\ M=P\left(L+\frac{x_{2}}{2}\right)\\ R=\sqrt{\frac{x_{2}^{2}}{4}+{\left(\frac{x_{1}+x_{3}}{2}\right)}^{2}}\\ J=2\left(\sqrt{2}x_{1}x_{2}\left(\frac{x_{2}^{2}}{12}+{\left(\frac{x_{1}+x_{3}}{2}\right)}^{2}\right)\right)\\ \sigma \left(x\right)=\frac{6PL}{x_{4}x_{3}^{2}}\\ \delta \left(x\right)=\frac{4PL^{3}}{Ex_{3}^{3}x_{4}}\\ P_{c}\left(x\right)=\frac{4.013E\sqrt{\frac{x_{3}^{2}x_{4}^{6}}{36}}}{L^{2}}\left(1-\frac{x_{3}}{2L}\sqrt{\frac{E}{4G}}\right)\\ P=6000\;lb\\ L=14\;in\\ \delta_{max}=0.25\;in\\ E=30\times 10^{6}\;psi\\ G=12\times 10^{6}\;psi\\ \tau_{max}=13600\;psi\\ \delta_{max}=30000\;psi \end{array}\right.$$

With bounds,

(38) $$0\leq x_{1},x_{4}\leq 2,$$

(39) $$0.1\leq x_{2},x_{3}\leq 10.$$

**Figure 20:**
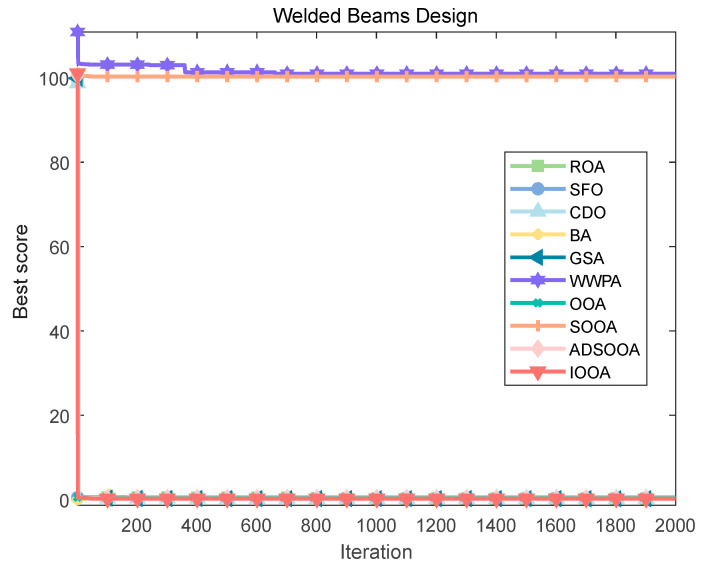
Iterative curves of various algorithms for the welded beams design.

To explore the solution of this optimization problem, several optimization algorithms were used for comparative evaluation. Figure 20 and Table 13 show that the IOOA algorithm stands out in this problem, and the optimal combination of design variables it finds achieves the minimization of the objective function value of 1.71 × 10^+00^, which is a more superior solution compared with the other algorithms, indicating that IOOA has higher efficiency and accuracy in solving this kind of complex structural optimization problems. This finding is not only of direct guidance to the engineering practice of welded beams but also provides a valuable reference for the application of optimization algorithms in the field of structural design.

### 5.3. Gear Train Optimization Design

**Figure 21:**
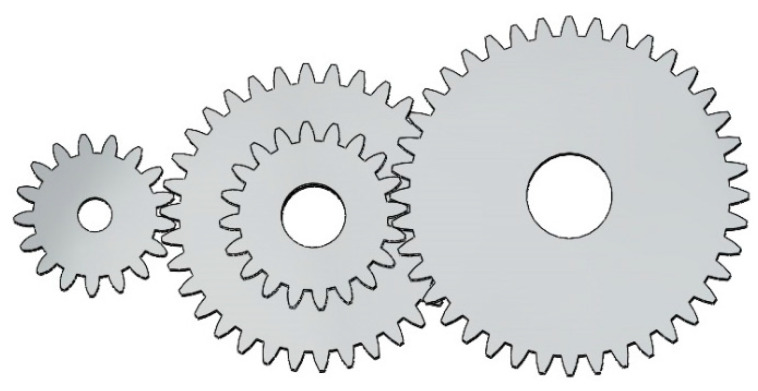
Simplified model of the gear train.

As a core component of mechanical engineering, the design optimization of gearing mechanisms is essential to improve equipment performance, reduce weight and size, and thus effectively control operating costs. This design challenge focuses on the construction of a precision transmission system with four gears, aiming to enhance the overall system efficiency by minimizing the transmission ratios, as shown in the simplified model in Figure 21.

The transmission ratio (gr) is quantified in this scenario as follows:

(40) $$minf\left(x\right)=(\frac{1}{6.931}-\frac{T_{D}T_{B}}{T_{A}T_{F}}{)}^{2}$$

where *TA*, *TB*, *TD*, and *TF* represent the number of teeth of the four gears, respectively, and the range of their values is bounded:

(41) $$s.t.\left\{\begin{array}{c} 12\leq T_{A}\leq 60\\ 12\leq T_{B}\leq 60\\ 12\leq T_{D}\leq 60\\ 12\leq T_{F}\leq 60 \end{array}\right.$$

**Figure 22:**
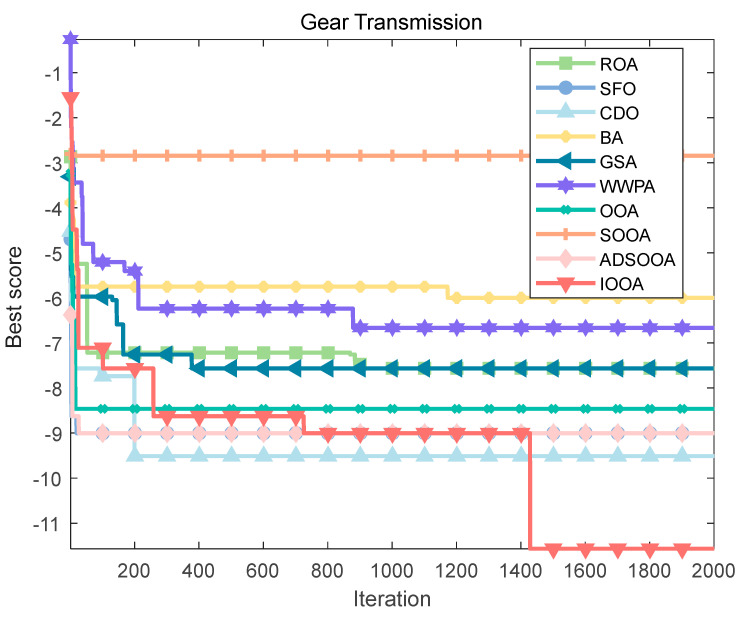
Iterative curves of various algorithms for gear transmission.

To explore the solution of this optimization problem, a series of optimization algorithms are employed, and their performances are compared. The results of the fitness iteration curves for each algorithm are shown in Figure 22, which shows that IOOA has a faster convergence speed and higher convergence accuracy than the other algorithms. Table 14 summarizes the optimal design variables and their corresponding minimum values of the objective function *f*min(*X*) obtained by each algorithm, and the results show that IOOA stands out with a significant advantage, with its computed objective function value as low as (2.70 × 10^−12^), which reveals that in the pursuit of a very small transmission ratio to improve the system efficiency, the IOOA algorithm is able to most accurately approximate the ideal solution, outperforming other algorithms such as ROA, SFO, CDO, BA, GSA, WWPA, OOA, SOOA, and ADSOOA. This finding not only emphasizes the efficiency and accuracy of the IOOA algorithm in solving complex gearing design problems but also provides a powerful tool for finding better gearing solutions in the field of mechanical design, which further promotes the development of high-performance gearing systems.

## 6. IOOA Performance Analysis and Conclusions

### 6.1. IOOA Performance Analysis

IOOA demonstrates excellent performance on several benchmark functions, especially showing fast convergence on the general test functions, CEC2020 and CEC2022 test sets. Specifically, IOOA significantly outperforms other algorithms in terms of convergence speed and accuracy on functions F1, F2, F6 to F8, F9, F12, F16, F18, F19, F24, F27, and F29, while on functions F3, F10, F11, F14, F15, F13, and F17, it maintains high convergence, despite convergence speeds that are similar to those of other algorithms’ accuracy. Statistical analysis of multiple independent runs of several test functions shows that IOOA performs well in terms of stability, with its minimum, mean, and standard deviation remaining low on most of the test functions, especially on functions such as F6, F8, F19, F26, and F30, indicating that it maintains good consistency of results while maintaining efficient optimization search. Boxplot analysis further confirms that IOOA shows stability with low median values and narrow distribution on most of the tested functions. By introducing the two-color complementary mechanism, IOOA effectively improves the balance in the search process and enables the algorithm to achieve a better balance between global search and local exploitation, which speeds up the convergence and improves the efficiency of resource utilization. Especially when dealing with test functions with high complexity, such as some functions in CEC2020 and CEC2022, IOOA is able to find near-optimal solutions in a shorter period of time, which reflects high efficiency. IOOA is able to find the optimal solution or near-optimal solutions on several test functions, especially on the functions F19, F24, F27, and F29, with the minimum value, mean, and standard deviation better than other algorithms, indicating its strong effectiveness in dealing with complex optimization problems. Through the Wilcoxon signed rank test and the Friedman test, IOOA shows significant performance advantages over other algorithms on most of the tested functions, which further validates its effectiveness. By introducing different policy adjustment mechanisms, IOOA ensures that the algorithm can accurately locate the global optimal solution or a near-optimal solution, especially on the CEC2020 and CEC2022 test sets. IOOA can find the minimum or near-minimum solution on most test functions, which fully proves its accuracy.

In summary, IOOA is able to quickly converge to the optimal solution or near-optimal solution on most of the test functions, demonstrating excellent convergence performance and, at the same time, demonstrating a high degree of stability and the ability to maintain consistent result quality. On the test functions with higher complexity, IOOA performs well and can effectively solve practical engineering problems. Although IOOA performs well on most of the test functions, it slightly underperforms other algorithms on some specific types of functions (e.g., F4, F11, F22, and F23), which may be due to the special nature of these functions. The convergence speed of IOOA may suffer when dealing with certain complex problems that are highly nonlinear or have multiple local optimal solutions. Overall, IOOA demonstrates strong capabilities in solving complex optimization problems, especially in terms of convergence speed, stability, and accuracy.

### 6.2. Conclusions

In this paper, in order to solve the shortcomings of OOA, that is, the search accuracy is not enough, and it is easy to fall into the local optimal solution, an innovative improvement strategy is proposed, and IOOA based on the two-color complementary mechanism is designed and implemented. By drawing on the four-color theory and multi-strategy fusion, the algorithm has achieved more superior performance in the field of global optimization. In the initialization stage of the algorithm, the good point set strategy of logistic chaotic mapping is used to construct an initial population with high diversity, and the four-color classification method is introduced to reconstruct the population, which effectively improves the global search ability of the algorithm. Subsequently, the core lies in the proposed two-color complementary mechanism, which distinguishes the roles and functions of the population through refinement. The blue and red populations, respectively, assume the roles of robust exploration and local obstacle avoidance, while the green and orange populations further enhance the randomness of the algorithm and the ability to effectively escape from the local optimal solution by introducing the following strategies: roaming and optimal spiral search. The embedding of the firefly perturbation strategy provides a new idea for solving local optimization problems. In the experimental part, this paper selects a series of classic benchmark test functions and the standard test sets of CEC2020 and CEC2022 to systematically evaluate the performance of IOOA and compare it in detail with various existing state-of-the-art optimization algorithms. Through rigorous verification of statistical tests (ANOVA, Wilcoxon test, and Friedman test), it was confirmed that IOOA has significantly improved convergence accuracy and convergence speed. At the same time, IOOA was applied to three engineering optimization design problems, indicating that the algorithm has a wider application potential and competitive advantage in solving complex optimization problems. In future research, we can further explore the integration of more diverse bio-inspired strategies with IOOA in order to achieve a deeper optimization effect in optimization problems in specific fields.

## Figures and Tables

**Table 1:** The list of internal parameters of algorithms.

Algorithm	Parameter
ROA	C=0.1;
SFO	PD=23;
CDO	$S_{\beta }=\log_{10}\left(rand\left(1:270,000\right)\right)$;
BA	A=0.5, r=0.5;
GSA	a=−pi, b=pi;
WWPA	$C=5Rand\left(1\right)$, $F=5Rand\left(1\right)$;
OOA	P=0.5;
SOOA	α=0;
ADSOOA	α=0.2, β=100;
IOOA	${\;p}_{0}=0.5,\delta =0.02$,${\;p}_{min}=0.2$;

**Table 2:** The information of benchmark test function.

No.	Function	dim	Range	F_min_
1	Rotated Ackley’s Function	100	[−100, 100]	0
2	Schwefel’s Problem 1.2 with Noise	100	[−50, 50]	0
3	Rotated Hybrid Composition Function with Noise	2	[−65.536, 65.536]	0
4	Hybrid Composition Function	4	[−5, 5]	0
5	Composition Function No. 4	6	[0, 1]	−3.32
6	BOOTH FUNCTION	2	[−10, 10]	0
7	ROSENBROCK FUNCTION	100	[−5, 10]	0
8	COLVILLE FUNCTION	4	[−10, 10]	0

**Table 3:** The information of CEC2020.

	No.	Functions	f_opt_
Unimodal function	9	Shifted and Rotated Bent Cigar Function	100
Basic functions	10	Shifted and Rotated Schwefel’s Function	1100
	11	Shifted and Rotated Lunacek bi-Rastrigin Function	700
	12	Expanded Rosenbrock’s plus Griewangk’s Function	1900
Hybrid functions	13	Hybrid Function 1 (N = 3)	1700
	14	Hybrid Function 2 (N = 4)	1600
	15	Hybrid Function 3 (N = 5)	2100
Composition functions	16	Composition Function 1 (N = 3)	2200
	17	Composition Function 2 (N = 4)	2400
	18	Composition Function 3 (N = 5)	2500
Search range: [−100, 100]			

**Table 4:** The information of CEC2022.

	No.	Functions	f_opt_
Unimodal function	19	Shifted and full-rotated Zakharov function	300
Basic functions	20	Shifted and full-rotated Rosenbrock’s function	400
	21	Shifted and full-rotated expanded Schaffer’s f6 function	600
	22	Shifted and full-rotated noncontinuous Rastrigin’s function	800
	23	Shifted and full-rotated levy function	900
Hybrid functions	24	Hybrid Function 1 (N = 3)	1800
	25	Hybrid Function 2 (N = 6)	2000
	26	Hybrid Function 3 (N = 5)	2200
Composition functions	27	Composition Function 1 (N = 5)	2300
	28	Composition Function 2 (N = 4)	2400
	29	Composition Function 3 (N = 5)	2600
	30	Composition Function 4 (N = 6)	2700
Search range: [−100, 100]			

**Table 5:** Experimental parameter setting.

Function	N	T	Dim
Benchmark functions	50	1000	[0, 100]
CEC2020	50	2000	20
CEC2022	50	10,000	20

**Table 6:** Experimental results on benchmark functions.

Function		ROA	SFO	CDO	BA	GSA	WWPA	OOA	SOOA	ADSOOA	IOOA
F1	Best	2.35 × 10^−06^	4.45 × 10^+00^	2.50 × 10^+01^	3.41 × 10^+04^	1.04 × 10^−06^	1.57 × 10^−01^	0.00 × 10^+00^	1.35 × 10^−02^	1.63 × 10^+00^	7.70 × 10^−30^
	Mean	1.68 × 10^−04^	1.50 × 10^+01^	2.50 × 10^+01^	5.49 × 10^+04^	1.57 × 10^−04^	5.77 × 10^+00^	1.45 × 10^−08^	7.94 × 10^−02^	4.75 × 10^+00^	3.68 × 10^−10^
	Std	1.40 × 10^−04^	5.85 × 10^+00^	0.00 × 10^+00^	1.34 × 10^+04^	3.92 × 10^−04^	5.79 × 10^+00^	4.57 × 10^−08^	3.25 × 10^−02^	4.58 × 10^+00^	1.16 × 10^−09^
	Iter	866	1000	557	1000	916	18	185	1000	475	976
F2	Best	1.00 × 10^−09^	3.10 × 10^−03^	1.16 × 10^+00^	3.19 × 10^+06^	6.67 × 10^−09^	6.74 × 10^−04^	4.71 × 10^−33^	7.90 × 10^−05^	2.10 × 10^−02^	4.71 × 10^−33^
	Mean	1.87 × 10^−06^	2.04 × 10^−01^	1.27 × 10^+00^	2.68 × 10^+07^	4.98 × 10^−07^	9.42 × 10^−02^	1.35 × 10^−13^	6.62 × 10^−04^	3.13 × 10^−02^	7.32 × 10^−15^
	Std	2.98 × 10^−06^	2.74 × 10^−01^	8.16 × 10^−02^	1.63 × 10^+07^	7.00 × 10^−07^	1.59 × 10^−01^	4.18 × 10^−13^	4.25 × 10^−04^	1.51 × 10^−02^	1.94 × 10^−14^
	Iter	918	985	848	1000	706	3	92	1000	249	731
F3	Best	9.98 × 10^−01^	1.99 × 10^+00^	9.90 × 10^+00^	2.98 × 10^+00^	9.98 × 10^−01^	9.98 × 10^−01^	9.98 × 10^−01^	9.98 × 10^−01^	9.98 × 10^−01^	9.98 × 10^−01^
	Mean	9.98 × 10^−01^	8.13 × 10^+00^	1.23 × 10^+01^	9.34 × 10^+00^	9.98 × 10^−01^	3.51 × 10^+00^	1.69 × 10^+00^	2.88 × 10^+00^	1.61 × 10^+00^	9.98 × 10^−01^
	Std	2.03 × 10^−11^	4.55 × 10^+00^	1.21 × 10^+00^	6.43 × 10^+00^	5.20 × 10^−10^	2.98 × 10^+00^	8.17 × 10^−01^	1.99 × 10^+00^	5.01 × 10^−01^	1.30 × 10^−15^
	Iter	572	945	1	183	504	220	342	326	871	906
F4	Best	3.11 × 10^−04^	3.31 × 10^−04^	3.12 × 10^−04^	1.02 × 10^−03^	3.12 × 10^−04^	1.82 × 10^−03^	3.12 × 10^−04^	4.01 × 10^−04^	3.07 × 10^−04^	3.08 × 10^−04^
	Mean	4.50 × 10^−04^	8.99 × 10^−03^	3.21 × 10^−04^	4.85 × 10^−03^	3.58 × 10^−04^	1.25 × 10^−02^	4.74 × 10^−04^	2.64 × 10^−03^	3.08 × 10^−04^	3.10 × 10^−04^
	Std	2.08 × 10^−04^	1.03 × 10^−02^	6.43 × 10^−06^	5.49 × 10^−03^	6.83 × 10^−05^	9.24 × 10^−03^	2.56 × 10^−04^	3.16 × 10^−03^	6.70 × 10^−07^	1.81 × 10^−06^
	Iter	650	619	504	999	918	303	456	1000	949	1000
F5	Best	−3.20 × 10^+00^	−3.23 × 10^+00^	−3.29 × 10^+00^	−3.32 × 10^+00^	−3.27 × 10^+00^	−1.83 × 10^+00^	−2.96 × 10^+00^	−3.09 × 10^+00^	−3.32 × 10^+00^	−3.32 × 10^+00^
	Mean	−2.95 × 10^+00^	−3.04 × 10^+00^	−3.23 × 10^+00^	−3.24 × 10^+00^	−3.11 × 10^+00^	−1.41 × 10^+00^	−2.52 × 10^+00^	−2.73 × 10^+00^	−3.25 × 10^+00^	−3.32 × 10^+00^
	Std	3.42 × 10^−01^	1.44 × 10^−01^	5.55 × 10^−02^	5.74 × 10^−02^	1.41 × 10^−01^	2.43 × 10^−01^	3.08 × 10^−01^	2.94 × 10^−01^	7.35 × 10^−02^	3.74 × 10^−06^
	Iter	685	982	168	824	922	234	155	67	996	997
F6	Best	4.31 × 10^−06^	3.05 × 10^−10^	3.43 × 10^−05^	1.41 × 10^−11^	7.95 × 10^−06^	1.20 × 10^−01^	6.25 × 10^−14^	2.35 × 10^−26^	3.83 × 10^−14^	0.00 × 10^+00^
	Mean	2.00 × 10^−01^	9.43 × 10^−09^	2.31 × 10^−04^	1.28 × 10^−10^	6.16 × 10^−05^	1.21 × 10^+00^	3.54 × 10^−03^	4.94 × 10^−04^	2.10 × 10^−06^	2.78 × 10^−16^
	Std	6.32 × 10^−01^	9.06 × 10^−09^	1.65 × 10^−04^	1.51 × 10^−10^	4.57 × 10^−05^	7.25 × 10^−01^	1.07 × 10^−02^	1.56 × 10^−03^	5.01 × 10^−06^	8.81 × 10^−16^
	Iter	598	785	524	321	789	67	149	340	912	765
F7	Best	1.61 × 10^−06^	4.85 × 10^+01^	4.69 × 10^+01^	4.24 × 10^+01^	1.62 × 10^−06^	9.33 × 10^−01^	0.00 × 10^+00^	4.85 × 10^+01^	4.84 × 10^+01^	1.23 × 10^−13^
	Mean	1.25 × 10^−04^	4.85 × 10^+01^	4.71 × 10^+01^	4.47 × 10^+01^	1.20 × 10^−04^	6.94 × 10^+01^	2.43 × 10^+01^	4.85 × 10^+01^	4.85 × 10^+01^	3.09 × 10^+01^
	Std	1.75 × 10^−04^	9.51 × 10^−04^	9.41 × 10^−02^	1.35 × 10^+00^	1.58 × 10^−04^	5.70 × 10^+01^	2.56 × 10^+01^	2.50 × 10^−03^	3.28 × 10^−02^	2.32 × 10^+01^
	Iter	652	578	308	1000	530	66	185	1000	935	982
F8	Best	2.03 × 10^−06^	9.18 × 10^−05^	2.27 × 10^−02^	4.54 × 10^−06^	1.97 × 10^−07^	2.13 × 10^−01^	0.00 × 10^+00^	6.94 × 10^−03^	2.84 × 10^−07^	0.00 × 10^+00^
	Mean	2.50 × 10^−04^	4.20 × 10^+00^	1.35 × 10^−01^	6.27 × 10^−01^	1.64 × 10^−05^	6.08 × 10^+00^	4.36 × 10^−05^	2.14 × 10^+00^	6.98 × 10^−03^	1.17 × 10^−13^
	Std	3.40 × 10^−04^	3.27 × 10^+00^	1.52 × 10^−01^	1.27 × 10^+00^	3.52 × 10^−05^	7.71 × 10^+00^	1.38 × 10^−04^	2.31 × 10^+00^	1.23 × 10^−02^	3.42 × 10^−13^
	Iter	648	464	218	992	705	13	766	1000	967	895

**Table 7:** Experimental results on CEC2020.

Function		ROA	SFO	CDO	BA	GSA	WWPA	OOA	SOOA	ADSOOA	IOOA
F9	Best	7.39 × 10^+09^	9.26 × 10^+09^	2.11 × 10^+10^	1.39 × 10^+10^	1.08 × 10^+10^	4.05 × 10^+10^	1.71 × 10^+10^	1.68 × 10^+10^	1.01 × 10^+10^	2.63 × 10^+05^
	Mean	1.93 × 10^+10^	1.88 × 10^+10^	2.12 × 10^+10^	2.37 × 10^+10^	1.75 × 10^+10^	4.28 × 10^+10^	2.60 × 10^+10^	2.40 × 10^+10^	1.47 × 10^+10^	5.58 × 10^+07^
	Std	4.87 × 10^+09^	6.44 × 10^+09^	1.40 × 10^+08^	7.38 × 10^+09^	5.85 × 10^+09^	1.20 × 10^+09^	6.91 × 10^+09^	4.24 × 10^+09^	2.50 × 10^+09^	1.16 × 10^+08^
	Iter	861	1943	697	2000	1953	911	462	572	1986	1999
F10	Best	4.10 × 10^+03^	4.79 × 10^+03^	4.35 × 10^+03^	3.46 × 10^+03^	4.63 × 10^+03^	5.83 × 10^+03^	4.54 × 10^+03^	4.70 × 10^+03^	3.79 × 10^+03^	2.80 × 10^+03^
	Mean	4.95 × 10^+03^	5.39 × 10^+03^	4.84 × 10^+03^	4.30 × 10^+03^	5.14 × 10^+03^	6.16 × 10^+03^	5.00 × 10^+03^	5.31 × 10^+03^	4.26 × 10^+03^	3.23 × 10^+03^
	Std	6.11 × 10^+02^	4.12 × 10^+02^	2.73 × 10^+02^	4.38 × 10^+02^	4.44 × 10^+02^	2.63 × 10^+02^	3.42 × 10^+02^	4.22 × 10^+02^	4.00 × 10^+02^	3.45 × 10^+02^
	Iter	766	610	493	1367	1529	226	1396	571	631	1998
F11	Best	9.44 × 10^+02^	1.00 × 10^+03^	9.77 × 10^+02^	1.07 × 10^+03^	1.01 × 10^+03^	1.11 × 10^+03^	9.81 × 10^+02^	9.50 × 10^+02^	8.99 × 10^+02^	8.81 × 10^+02^
	Mean	9.95 × 10^+02^	1.03 × 10^+03^	9.85 × 10^+02^	1.23 × 10^+03^	1.04 × 10^+03^	1.13 × 10^+03^	1.02 × 10^+03^	1.03 × 10^+03^	9.44 × 10^+02^	9.42 × 10^+02^
	Std	2.79 × 10^+01^	1.97 × 10^+01^	1.26 × 10^+01^	1.11 × 10^+02^	1.66 × 10^+01^	1.11 × 10^+01^	2.15 × 10^+01^	3.42 × 10^+01^	3.28 × 10^+01^	3.15 × 10^+01^
	Iter	921	1814	761	1394	2000	55	333	873	151	2000
F12	Best	1.70 × 10^+04^	3.28 × 10^+04^	3.87 × 10^+05^	2.42 × 10^+04^	5.68 × 10^+03^	1.71 × 10^+06^	9.21 × 10^+04^	2.48 × 10^+04^	1.57 × 10^+04^	1.91 × 10^+03^
	Mean	3.37 × 10^+05^	1.04 × 10^+05^	3.88 × 10^+05^	5.37 × 10^+05^	1.23 × 10^+05^	2.86 × 10^+06^	5.15 × 10^+05^	1.60 × 10^+05^	1.09 × 10^+05^	1.93 × 10^+03^
	Std	8.02 × 10^+05^	6.25 × 10^+04^	7.95 × 10^+02^	6.04 × 10^+05^	8.43 × 10^+04^	9.13 × 10^+05^	3.46 × 10^+05^	1.11 × 10^+05^	1.21 × 10^+05^	2.02 × 10^+01^
	Iter	840	1664	482	2000	1978	83	1167	456	1771	1699
F13	Best	9.72 × 10^+05^	5.99 × 10^+05^	4.16 × 10^+05^	2.39 × 10^+05^	1.20 × 10^+06^	6.53 × 10^+06^	3.33 × 10^+06^	1.60 × 10^+06^	2.86 × 10^+05^	1.64 × 10^+05^
	Mean	2.75 × 10^+06^	3.58 × 10^+06^	6.69 × 10^+05^	1.03 × 10^+07^	3.41 × 10^+06^	2.69 × 10^+07^	6.30 × 10^+06^	6.35 × 10^+06^	9.83 × 10^+05^	7.77 × 10^+05^
	Std	1.37 × 10^+06^	2.32 × 10^+06^	2.72 × 10^+05^	1.50 × 10^+07^	1.51 × 10^+06^	9.89 × 10^+06^	3.04 × 10^+06^	5.56 × 10^+06^	6.25 × 10^+05^	4.19 × 10^+05^
	Iter	1270	1082	787	2000	1788	70	1898	260	1951	1986
F14	Best	2.39 × 10^+03^	2.36 × 10^+03^	2.76 × 10^+03^	2.64 × 10^+03^	2.61 × 10^+03^	3.49 × 10^+03^	2.81 × 10^+03^	2.67 × 10^+03^	2.16 × 10^+03^	2.11 × 10^+03^
	Mean	2.74 × 10^+03^	3.02 × 10^+03^	3.09 × 10^+03^	3.05 × 10^+03^	3.02 × 10^+03^	4.17 × 10^+03^	3.04 × 10^+03^	3.18 × 10^+03^	2.57 × 10^+03^	2.37 × 10^+03^
	Std	2.88 × 10^+02^	3.59 × 10^+02^	1.82 × 10^+02^	2.94 × 10^+02^	3.20 × 10^+02^	3.49 × 10^+02^	1.55 × 10^+02^	2.37 × 10^+02^	2.57 × 10^+02^	2.34 × 10^+02^
	Iter	910	1993	443	2000	1865	181	1990	1888	1976	1727
F15	Best	1.28 × 10^+05^	6.66 × 10^+04^	3.45 × 10^+05^	4.24 × 10^+06^	1.09 × 10^+05^	5.72 × 10^+06^	1.91 × 10^+05^	3.19 × 10^+05^	1.40 × 10^+05^	5.45 × 10^+04^
	Mean	1.25 × 10^+06^	3.54 × 10^+06^	2.42 × 10^+07^	7.99 × 10^+06^	2.21 × 10^+06^	2.86 × 10^+07^	1.86 × 10^+06^	1.72 × 10^+06^	4.30 × 10^+05^	3.51 × 10^+05^
	Std	1.74 × 10^+06^	2.85 × 10^+06^	2.59 × 10^+07^	5.86 × 10^+06^	2.31 × 10^+06^	2.24 × 10^+07^	2.35 × 10^+06^	1.29 × 10^+06^	2.57 × 10^+05^	3.32 × 10^+05^
	Iter	1331	1735	916	2000	1971	28	1603	1998	1883	2000
F16	Best	3.78 × 10^+03^	3.42 × 10^+03^	4.22 × 10^+03^	4.19 × 10^+03^	3.66 × 10^+03^	6.71 × 10^+03^	3.88 × 10^+03^	4.43 × 10^+03^	3.37 × 10^+03^	2.31 × 10^+03^
	Mean	5.24 × 10^+03^	5.51 × 10^+03^	5.22 × 10^+03^	5.82 × 10^+03^	5.67 × 10^+03^	7.41 × 10^+03^	5.33 × 10^+03^	5.55 × 10^+03^	3.90 × 10^+03^	2.32 × 10^+03^
	Std	1.16 × 10^+03^	1.61 × 10^+03^	1.31 × 10^+03^	8.62 × 10^+02^	9.65 × 10^+02^	2.78 × 10^+02^	9.29 × 10^+02^	6.49 × 10^+02^	3.38 × 10^+02^	1.22 × 10^+01^
	Iter	613	1527	1370	2000	1711	222	1012	1994	1976	1986
F17	Best	3.08 × 10^+03^	2.95 × 10^+03^	3.24 × 10^+03^	3.14 × 10^+03^	3.05 × 10^+03^	3.20 × 10^+03^	3.28 × 10^+03^	3.34 × 10^+03^	2.97 × 10^+03^	2.90 × 10^+03^
	Mean	3.19 × 10^+03^	3.17 × 10^+03^	3.34 × 10^+03^	3.27 × 10^+03^	3.25 × 10^+03^	3.48 × 10^+03^	3.55 × 10^+03^	3.53 × 10^+03^	3.01 × 10^+03^	3.03 × 10^+03^
	Std	7.99 × 10^+01^	1.06 × 10^+02^	4.93 × 10^+01^	8.12 × 10^+01^	1.87 × 10^+02^	2.07 × 10^+02^	1.78 × 10^+02^	1.20 × 10^+02^	2.09 × 10^+01^	1.13 × 10^+02^
	Iter	716	1993	443	2000	1997	80	170	97	1966	2000
F18	Best	3.44 × 10^+03^	3.63 × 10^+03^	5.91 × 10^+03^	4.24 × 10^+03^	3.28 × 10^+03^	6.85 × 10^+03^	4.41 × 10^+03^	3.95 × 10^+03^	3.34 × 10^+03^	2.96 × 10^+03^
	Mean	4.20 × 10^+03^	4.31 × 10^+03^	5.92 × 10^+03^	5.46 × 10^+03^	4.51 × 10^+03^	8.72 × 10^+03^	5.59 × 10^+03^	4.90 × 10^+03^	4.01 × 10^+03^	3.00 × 10^+03^
	Std	7.69 × 10^+02^	6.30 × 10^+02^	8.74 × 10^+00^	1.48 × 10^+03^	6.59 × 10^+02^	1.34 × 10^+03^	9.61 × 10^+02^	6.74 × 10^+02^	3.16 × 10^+02^	2.43 × 10^+01^
	Iter	977	1993	591	2000	1920	94	358	717	673	2000

**Table 8:** Experimental results on CEC2022.

Function		ROA	SFO	CDO	BA	GSA	WWPA	OOA	SOOA	ADSOOA	IOOA
F19	Best	1.52 × 10^+04^	2.74 × 10^+04^	2.25 × 10^+04^	2.37 × 10^+03^	2.08 × 10^+04^	9.28 × 10^+04^	2.11 × 10^+04^	2.65 × 10^+04^	1.13 × 10^+04^	3.00 × 10^+02^
	Mean	3.58 × 10^+04^	4.75 × 10^+04^	2.26 × 10^+04^	1.13 × 10^+05^	4.82 × 10^+04^	1.56 × 10^+05^	4.17 × 10^+04^	5.16 × 10^+04^	1.76 × 10^+04^	3.01 × 10^+02^
	Std	1.35 × 10^+04^	1.31 × 10^+04^	6.16 × 10^+01^	1.26 × 10^+05^	4.00 × 10^+04^	3.65 × 10^+04^	8.34 × 10^+03^	2.61 × 10^+04^	3.93 × 10^+03^	2.46 × 10^+00^
	Iter	5768	9929	2853	10,000	7392	630	596	486	7884	10,000
F20	Best	8.51 × 10^+02^	1.14 × 10^+03^	1.96 × 10^+03^	6.44 × 10^+02^	8.63 × 10^+02^	2.11 × 10^+03^	1.59 × 10^+03^	1.04 × 10^+03^	1.11 × 10^+03^	4.03 × 10^+02^
	Mean	1.42 × 10^+03^	1.71 × 10^+03^	1.97 × 10^+03^	8.34 × 10^+02^	1.46 × 10^+03^	4.11 × 10^+03^	2.50 × 10^+03^	2.06 × 10^+03^	1.47 × 10^+03^	4.33 × 10^+02^
	Std	5.07 × 10^+02^	4.22 × 10^+02^	5.07 × 10^+00^	1.35 × 10^+02^	4.72 × 10^+02^	1.11 × 10^+03^	6.13 × 10^+02^	6.50 × 10^+02^	3.07 × 10^+02^	1.58 × 10^+01^
	Iter	5137	9966	3955	10,000	8320	86	1382	5893	5387	9975
F21	Best	6.47 × 10^+02^	6.63 × 10^+02^	6.47 × 10^+02^	6.59 × 10^+02^	6.64 × 10^+02^	6.98 × 10^+02^	6.53 × 10^+02^	6.59 × 10^+02^	6.58 × 10^+02^	6.41 × 10^+02^
	Mean	6.68 × 10^+02^	6.81 × 10^+02^	6.49 × 10^+02^	6.67 × 10^+02^	6.78 × 10^+02^	7.10 × 10^+02^	6.71 × 10^+02^	6.72 × 10^+02^	6.66 × 10^+02^	6.57 × 10^+02^
	Std	1.24 × 10^+01^	1.02 × 10^+01^	1.93 × 10^+00^	9.12 × 10^+00^	1.08 × 10^+01^	6.71 × 10^+00^	1.12 × 10^+01^	8.48 × 10^+00^	5.38 × 10^+00^	6.86 × 10^+00^
	Iter	4257	9900	2824	10,000	9376	455	6241	7017	6145	9883
F22	Best	9.07 × 10^+02^	9.63 × 10^+02^	8.99 × 10^+02^	8.62 × 10^+02^	9.16 × 10^+02^	9.96 × 10^+02^	9.34 × 10^+02^	9.43 × 10^+02^	9.19 × 10^+02^	8.72 × 10^+02^
	Mean	9.43 × 10^+02^	9.86 × 10^+02^	9.19 × 10^+02^	8.91 × 10^+02^	9.42 × 10^+02^	1.02 × 10^+03^	9.62 × 10^+02^	9.65 × 10^+02^	9.37 × 10^+02^	8.86 × 10^+02^
	Std	1.70 × 10^+01^	1.71 × 10^+01^	1.37 × 10^+01^	2.37 × 10^+01^	1.71 × 10^+01^	1.14 × 10^+01^	1.30 × 10^+01^	1.72 × 10^+01^	1.48 × 10^+01^	7.08 × 10^+00^
	Iter	4598	3753	3868	3229	8730	3682	1316	2447	9418	9779
F23	Best	2.59 × 10^+03^	2.76 × 10^+03^	2.76 × 10^+03^	2.43 × 10^+03^	2.94 × 10^+03^	5.09 × 10^+03^	2.80 × 10^+03^	2.53 × 10^+03^	2.36 × 10^+03^	2.39 × 10^+03^
	Mean	3.27 × 10^+03^	4.28 × 10^+03^	2.89 × 10^+03^	3.26 × 10^+03^	3.31 × 10^+03^	5.47 × 10^+03^	3.37 × 10^+03^	3.08 × 10^+03^	2.76 × 10^+03^	2.43 × 10^+03^
	Std	4.41 × 10^+02^	7.97 × 10^+02^	7.30 × 10^+01^	6.84 × 10^+02^	1.94 × 10^+02^	2.28 × 10^+02^	4.23 × 10^+02^	4.61 × 10^+02^	2.61 × 10^+02^	1.41 × 10^+01^
	Iter	8304	9688	4073	9514	9373	79	9892	5547	5135	9994
F24	Best	4.95 × 10^+06^	7.87 × 10^+07^	7.28 × 10^+08^	2.18 × 10^+03^	2.95 × 10^+07^	2.95 × 10^+09^	1.57 × 10^+08^	5.38 × 10^+08^	6.15 × 10^+07^	2.01 × 10^+03^
	Mean	4.00 × 10^+08^	2.25 × 10^+08^	3.34 × 10^+09^	8.65 × 10^+03^	6.43 × 10^+08^	6.09 × 10^+09^	1.56 × 10^+09^	1.16 × 10^+09^	3.22 × 10^+08^	3.24 × 10^+03^
	Std	5.73 × 10^+08^	1.13 × 10^+08^	2.75 × 10^+09^	9.68 × 10^+03^	7.00 × 10^+08^	1.71 × 10^+09^	1.03 × 10^+09^	7.46 × 10^+08^	2.36 × 10^+08^	6.82 × 10^+02^
	Iter	4646	9960	4142	9818	9803	112	1119	1565	9700	6264
F25	Best	2.11 × 10^+03^	2.17 × 10^+03^	2.26 × 10^+03^	2.16 × 10^+03^	2.16 × 10^+03^	2.35 × 10^+03^	2.15 × 10^+03^	2.16 × 10^+03^	2.12 × 10^+03^	2.07 × 10^+03^
	Mean	2.18 × 10^+03^	2.31 × 10^+03^	2.30 × 10^+03^	2.28 × 10^+03^	2.22 × 10^+03^	2.45 × 10^+03^	2.18 × 10^+03^	2.21 × 10^+03^	2.15 × 10^+03^	2.16 × 10^+03^
	Std	4.55 × 10^+01^	1.04 × 10^+02^	3.57 × 10^+01^	9.88 × 10^+01^	4.21 × 10^+01^	5.81 × 10^+01^	1.84 × 10^+01^	3.65 × 10^+01^	2.93 × 10^+01^	3.23 × 10^+01^
	Iter	4521	8979	3745	9478	9733	1770	909	10,000	9766	9309
F26	Best	2.24 × 10^+03^	2.26 × 10^+03^	2.24 × 10^+03^	2.61 × 10^+03^	2.23 × 10^+03^	2.34 × 10^+03^	2.25 × 10^+03^	2.24 × 10^+03^	2.23 × 10^+03^	2.23 × 10^+03^
	Mean	2.31 × 10^+03^	2.51 × 10^+03^	2.24 × 10^+03^	2.98 × 10^+03^	2.28 × 10^+03^	2.69 × 10^+03^	2.31 × 10^+03^	2.38 × 10^+03^	2.32 × 10^+03^	2.24 × 10^+03^
	Std	2.06 × 10^+02^	2.08 × 10^+02^	4.97 × 10^+00^	4.28 × 10^+02^	6.85 × 10^+01^	2.38 × 10^+02^	6.44 × 10^+01^	1.47 × 10^+02^	8.91 × 10^+01^	5.57 × 10^+00^
	Iter	5671	9894	3469	9476	9372	155	9941	3908	9486	10,000
F27	Best	2.73 × 10^+03^	2.77 × 10^+03^	3.08 × 10^+03^	2.64 × 10^+03^	2.65 × 10^+03^	3.81 × 10^+03^	2.96 × 10^+03^	3.03 × 10^+03^	2.64 × 10^+03^	2.47 × 10^+03^
	Mean	2.79 × 10^+03^	2.91 × 10^+03^	3.43 × 10^+03^	2.75 × 10^+03^	2.89 × 10^+03^	4.26 × 10^+03^	3.41 × 10^+03^	3.28 × 10^+03^	2.76 × 10^+03^	2.47 × 10^+03^
	Std	6.51 × 10^+01^	1.02 × 10^+02^	1.22 × 10^+02^	1.09 × 10^+02^	1.73 × 10^+02^	2.62 × 10^+02^	4.15 × 10^+02^	2.33 × 10^+02^	1.03 × 10^+02^	1.62 × 10^+00^
	Iter	7735	9993	3444	10,000	9468	262	2117	177	7694	9000
F28	Best	5.30 × 10^+03^	6.32 × 10^+03^	5.02 × 10^+03^	4.53 × 10^+03^	2.60 × 10^+03^	6.68 × 10^+03^	4.88 × 10^+03^	2.70 × 10^+03^	2.74 × 10^+03^	2.50 × 10^+03^
	Mean	5.88 × 10^+03^	7.15 × 10^+03^	5.55 × 10^+03^	5.24 × 10^+03^	5.56 × 10^+03^	7.17 × 10^+03^	6.40 × 10^+03^	5.27 × 10^+03^	4.23 × 10^+03^	2.77 × 10^+03^
	Std	4.26 × 10^+02^	4.54 × 10^+02^	3.47 × 10^+02^	5.16 × 10^+02^	1.11 × 10^+03^	2.83 × 10^+02^	6.32 × 10^+02^	1.73 × 10^+03^	1.20 × 10^+03^	5.77 × 10^+02^
	Iter	6587	9964	714	10,000	9688	4015	5790	9818	9124	9548
F29	Best	5.58 × 10^+03^	7.78 × 10^+03^	8.42 × 10^+03^	3.42 × 10^+04^	6.65 × 10^+03^	9.19 × 10^+03^	8.01 × 10^+03^	6.91 × 10^+03^	6.45 × 10^+03^	2.61 × 10^+03^
	Mean	7.16 × 10^+03^	8.61 × 10^+03^	8.44 × 10^+03^	5.33 × 10^+04^	7.72 × 10^+03^	9.76 × 10^+03^	8.92 × 10^+03^	8.34 × 10^+03^	7.41 × 10^+03^	2.77 × 10^+03^
	Std	9.07 × 10^+02^	6.37 × 10^+02^	1.85 × 10^+01^	1.31 × 10^+04^	5.50 × 10^+02^	3.60 × 10^+02^	5.51 × 10^+02^	6.47 × 10^+02^	6.89 × 10^+02^	1.68 × 10^+02^
	Iter	4764	4097	1620	10,000	9456	620	5484	110	5022	9995
F30	Best	3.04 × 10^+03^	3.07 × 10^+03^	3.44 × 10^+03^	2.90 × 10^+03^	3.15 × 10^+03^	2.90 × 10^+03^	3.65 × 10^+03^	3.64 × 10^+03^	3.11 × 10^+03^	2.98 × 10^+03^
	Mean	3.21 × 10^+03^	3.26 × 10^+03^	3.47 × 10^+03^	2.90 × 10^+03^	3.41 × 10^+03^	2.90 × 10^+03^	3.86 × 10^+03^	3.90 × 10^+03^	3.17 × 10^+03^	3.04 × 10^+03^
	Std	1.75 × 10^+02^	1.14 × 10^+02^	1.72 × 10^+01^	5.07 × 10^−05^	2.58 × 10^+02^	3.23 × 10^−05^	1.28 × 10^+02^	3.05 × 10^+02^	7.55 × 10^+01^	3.94 × 10^+01^
	Iter	4261	8365	3121	8418	5579	7426	402	9989	9943	9742

**Table 9:** The Wilcoxon test results on benchmark functions.

IOOA vs.	ROA	SFO	CDO	BA	GSA	WWPA	OOA	SOOA	ADSOOA
F1	0.0002 (+)	0.0002 (+)	0.0001 (+)	0.0002 (+)	0.0002 (+)	0.0002 (+)	0.5204 (=)	0.0002 (+)	0.0002 (+)
F2	0.0002 (+)	0.0002 (+)	0.0001 (+)	0.0002 (+)	0.0002 (+)	0.0002 (+)	0.4272 (=)	0.0002 (+)	0.0002 (+)
F3	NaN (=)	0.0001 (+)	0.0000 (+)	0.0001 (+)	NaN (=)	0.0008 (+)	0.0146 (+)	0.0007 (+)	0.0002 (+)
F4	0.0004 (+)	0.0002 (+)	0.0003 (+)	0.0002 (+)	0.0003 (+)	0.0002 (+)	0.0003 (+)	0.0002 (+)	0.0234 (−)
F5	0.0001 (+)	0.0001 (+)	0.0001 (+)	0.0016 (+)	0.0001 (+)	0.0001 (+)	0.0001 (+)	0.0001 (+)	0.0148 (+)
F6	0.0001 (+)	0.0001 (+)	0.0001 (+)	0.0001 (+)	0.0001 (+)	0.0001 (+)	0.0001 (+)	0.0009 (+)	0.0001 (+)
F7	0.1390 (=)	0.0060 (+)	0.4649 (=)	0.4708 (=)	0.1390 (=)	0.0252 (+)	0.4988 (=)	0.0060 (+)	0.0362 (+)
F8	0.0002 (+)	0.0002 (+)	0.0002 (+)	0.0002 (+)	0.0002 (+)	0.0002 (+)	0.5199 (=)	0.0002 (+)	0.0002 (+)
+/=/−	6/2/0	8/0/0	7/1/0	7/1/0	6/2/0	8/0/0	4/4/0	8/0/0	7/1/0

**Table 10:** The Wilcoxon test results on CEC2020.

IOOA vs.	ROA	SFO	CDO	BA	GSA	WWPA	OOA	SOOA	ADSOOA
F9	0.0002 (+)	0.0002 (+)	0.0002 (+)	0.0002 (+)	0.0002 (+)	0.0002 (+)	0.0002 (+)	0.0002 (+)	0.0002 (+)
F10	0.0002 (+)	0.0002 (+)	0.0002 (+)	0.0006 (+)	0.0002 (+)	0.0002 (+)	0.0002 (+)	0.0002 (+)	0.0002 (+)
F11	0.0028 (+)	0.0002 (+)	0.0002 (+)	0.0002 (+)	0.0002 (+)	0.0002 (+)	0.0002 (+)	0.0010 (+)	0.8501 (=)
F12	0.0002 (+)	0.0002 (+)	0.0002 (+)	0.0002 (+)	0.0002 (+)	0.0002 (+)	0.0002 (+)	0.0002 (+)	0.0002 (+)
F13	0.0010 (+)	0.0046 (+)	0.4960 (=)	0.0022 (+)	0.0002 (+)	0.0002 (+)	0.0002 (+)	0.0002 (+)	0.5964 (=)
F14	0.0081 (+)	0.0008 (+)	0.0002 (+)	0.0004 (+)	0.0010 (+)	0.0002 (+)	0.0002 (+)	0.0003 (+)	0.0694 (=)
F15	0.1041 (=)	0.0046 (+)	0.0013 (+)	0.0002 (+)	0.0091 (+)	0.0002 (+)	0.0073 (+)	0.0010 (+)	0.3075 (=)
F16	0.0002 (+)	0.0002 (+)	0.0002 (+)	0.0002 (+)	0.0002 (+)	0.0002 (+)	0.0002 (+)	0.0002 (+)	0.0002 (+)
F17	0.0091 (+)	0.0140 (+)	0.0002 (+)	0.0008 (+)	0.0058 (+)	0.0002 (+)	0.0002 (+)	0.0002 (+)	0.8501 (=)
F18	0.0002 (+)	0.0002 (+)	0.0002 (+)	0.0002 (+)	0.0002 (+)	0.0002 (+)	0.0002 (+)	0.0002 (+)	0.0002 (+)
+/=/−	9/1/0	10/0/0	9/1/0	10/0/0	10/0/0	10/0/0	10/0/0	10/0/0	5/5/0

**Table 11:** The Wilcoxon test results on CEC2022.

IOOA vs.	ROA	SFO	CDO	BA	GSA	WWPA	OOA	SOOA	ADSOOA
F19	0.0002 (+)	0.0002 (+)	0.0002 (+)	0.0002 (+)	0.0002 (+)	0.0002 (+)	0.0002 (+)	0.0002 (+)	0.0002 (+)
F20	0.0001 (+)	0.0001 (+)	0.0001 (+)	0.0001 (+)	0.0001 (+)	0.0001 (+)	0.0001 (+)	0.0001 (+)	0.0001 (+)
F21	0.0757 (=)	0.0002 (+)	0.0073 (-)	0.0140 (+)	0.0002 (+)	0.0002 (+)	0.0113 (+)	0.0017 (+)	0.0046 (+)
F22	0.0002 (+)	0.0002 (+)	0.0002 (+)	0.6232 (=)	0.0002 (+)	0.0002 (+)	0.0002 (+)	0.0002 (+)	0.0002 (+)
F23	0.0002 (+)	0.0002 (+)	0.0002 (+)	0.0010 (+)	0.0002 (+)	0.0002 (+)	0.0002 (+)	0.0002 (+)	0.0211 (+)
F24	0.0002 (+)	0.0002 (+)	0.0002 (+)	0.0539 (=)	0.0002 (+)	0.0002 (+)	0.0002 (+)	0.0002 (+)	0.0002 (+)
F25	0.4727 (=)	0.0008 (+)	0.0002 (+)	0.0140 (+)	0.0036 (+)	0.0002 (+)	0.0757 (=)	0.0046 (+)	0.1405 (=)
F26	0.0073 (+)	0.0002 (+)	0.0091 (+)	0.0002 (+)	0.0073 (+)	0.0002 (+)	0.0002 (+)	0.0003 (+)	0.0022 (+)
F27	0.0002 (+)	0.0002 (+)	0.0002 (+)	0.0002 (+)	0.0002 (+)	0.0002 (+)	0.0002 (+)	0.0002 (+)	0.0002 (+)
F28	0.0002 (+)	0.0002 (+)	0.0002 (+)	0.0002 (+)	0.0003 (+)	0.0002 (+)	0.0002 (+)	0.0010 (+)	0.0022 (+)
F29	0.0002 (+)	0.0002 (+)	0.0002 (+)	0.0002 (+)	0.0002 (+)	0.0002 (+)	0.0002 (+)	0.0002 (+)	0.0002 (+)
F30	0.0004 (+)	0.0003 (+)	0.0002 (+)	0.0001 (-)	0.0002 (+)	0.0002 (−)	0.0002 (+)	0.0002 (+)	0.0004 (+)
+/=/−	10/2/0	12/0/0	11/0/1	9/2/1	12/0/0	11/0/1	11/1/0	12/0/0	11/1/0

**Table 12:** Comparison results of the tension spring design.

Methods	Optimal Solution of Design Variables			*f*min (*X*)	Iter
	$x_{1}$	$x_{2}$	$x_{3}$		
ROA	5.89 × 10^−02^	5.54 × 10^−01^	5.09 × 10^+00^	1.36 × 10^−02^	1353
SFO	5.70 × 10^−02^	4.97 × 10^−01^	6.15 × 10^+00^	1.31 × 10^−02^	1418
CDO	5.00 × 10^−02^	3.17 × 10^−01^	1.42 × 10^+01^	1.28 × 10^−02^	904
BA	4.96 × 10^−02^	3.09 × 10^−01^	1.47 × 10^+01^	1.27 × 10^−02^	1638
GSA	5.85 × 10^−02^	5.44 × 10^−01^	5.30 × 10^+00^	1.36 × 10^−02^	2000
WWPA	1.44 × 10^−01^	1.30 × 10^+00^	1.50 × 10^+01^	3.99 × 10^+99^	185
OOA	5.37 × 10^−02^	4.61 × 10^−01^	4.20 × 10^+00^	1.1 × 10^+100^	276
SOOA	5.90 × 10^−02^	5.59 × 10^−01^	4.97 × 10^+00^	1.36 × 10^−02^	1442
ADSOOA	5.00 × 10^−02^	3.17 × 10^−01^	1.40 × 10^+01^	1.27 × 10^−02^	1870
IOOA	5.18 × 10^−02^	3.60 × 10^−01^	1.11 × 10^+01^	1.27 × 10^−02^	1247

**Table 13:** Comparison results of the welded beams design.

Methods	Optimal Solution of Design Variables				*f*min (*X*)	Iter
	$x_{1}$	$x_{2}$	$x_{3}$	$x_{4}$		
ROA	1.85 × 10^−01^	3.86 × 10^+00^	8.84 × 10^+00^	2.15 × 10^−01^	1.78 × 10^+00^	1334
SFO	1.39 × 10^−01^	7.69 × 10^+00^	7.14 × 10^+00^	3.29 × 10^−01^	2.62 × 10^+00^	1954
CDO	2.00 × 10^−01^	3.36 × 10^+00^	9.32 × 10^+00^	2.07 × 10^−01^	1.76 × 10^+00^	457
BA	−9.26 × 10^−02^	9.08 × 10^+00^	6.94 × 10^+00^	−3.28 × 10^−01^	−1.39 × 10^+108^	4
GSA	2.04 × 10^−01^	3.40 × 10^+00^	9.08 × 10^+00^	2.06 × 10^−01^	1.72 × 10^+00^	2000
WWPA	7.75 × 10^−01^	1.72 × 10^+00^	1.00 × 10^+01^	6.40 × 10^−01^	9.86 × 10^+100^	661
OOA	2.45 × 10^−01^	7.14 × 10^+00^	6.73 × 10^+00^	3.70 × 10^−01^	3.01 × 10^+00^	693
SOOA	1.73 × 10^+00^	5.62 × 10^−01^	3.32 × 10^+00^	1.52 × 10^+00^	2.08 × 10^+100^	501
ADSOOA	2.54 × 10^−01^	3.05 × 10^+00^	7.59 × 10^+00^	2.91 × 10^−01^	2.03 × 10^+00^	1974
IOOA	1.98 × 10^−01^	3.43 × 10^+00^	9.03 × 10^+00^	2.06 × 10^−01^	1.71 × 10^+00^	1997

**Table 14:** Comparison results of design problems of gear transmission.

Methods	Optimal Solution of Design Variables				*f*min (*X*)	Iter
	$x_{1}$	$x_{2}$	$x_{3}$	$x_{4}$		
ROA	6.00 × 10^+01^	2.02 × 10^+01^	2.62 × 10^+01^	6.00 × 10^+01^	2.73 × 10^−08^	915
SFO	4.66 × 10^+01^	1.31 × 10^+01^	1.21 × 10^+01^	2.30 × 10^+01^	9.92 × 10^−10^	20
CDO	3.73 × 10^+01^	2.11 × 10^+01^	1.45 × 10^+01^	5.86 × 10^+01^	3.07 × 10^−10^	200
BA	2.32 × 10^+02^	−2.05 × 10^+02^	−7.33 × 10^+01^	4.44 × 10^+02^	1.00 × 10^−06^	1173
GSA	6.00 × 10^+01^	1.35 × 10^+01^	4.03 × 10^+01^	6.00 × 10^+01^	2.73 × 10^−08^	2000
WWPA	2.92 × 10^+16^	1.23 × 10^+17^	1.21 × 10^+16^	3.53 × 10^+17^	2.17 × 10^−07^	879
OOA	4.12 × 10^+01^	1.63 × 10^+01^	1.67 × 10^+01^	4.56 × 10^+01^	3.45 × 10^−09^	16
SOOA	4.57 × 10^+01^	3.75 × 10^+01^	1.24 × 10^+01^	5.28 × 10^+01^	1.43 × 10^−03^	15
ADSOOA	2.33 × 10^+01^	1.33 × 10^+01^	1.20 × 10^+01^	4.66 × 10^+01^	9.92 × 10^−10^	27
IOOA	4.27 × 10^+01^	1.60 × 10^+01^	1.91 × 10^+01^	4.90 × 10^+01^	2.70 × 10^−12^	1429

## Data Availability

The data used in the study can be accessed upon your request to the author.
